# A Statistical Shape Modeling Approach for the Derivation of a Data‐Driven Geometry‐Aware Lumped Arterial Stenosis Model

**DOI:** 10.1002/cnm.70138

**Published:** 2026-02-05

**Authors:** P. L. J. Hilhorst, S. C. F. P. M. Verstraeten, K. Zając, R. Ganesan, M. van 't Veer, F. N. van de Vosse, W. Huberts

**Affiliations:** ^1^ Department of Biomedical Engineering Eindhoven University of Technology Eindhoven the Netherlands; ^2^ Sano Centre for Computational Medicine Kraków Poland; ^3^ Department of Cardiology Catharina Hospital Eindhoven the Netherlands; ^4^ Computational Science Lab, Faculty of Science Institute for Informatics, University of Amsterdam Amsterdam the Netherlands

**Keywords:** computational fluid dynamics, coronary hemodynamics, pressure drop prediction, reduced‐order modeling, statistical shape modeling

## Abstract

Existing lumped arterial stenosis models struggle to accurately capture the pressure and flow relationship of complex lesion morphologies, thereby limiting their ability to accurately evaluate lesions. To overcome these limitations, we introduce a geometry‐informed, data‐driven lumped stenosis model that incorporates realistic lesion shapes using statistical shape modeling (SSM). By generating a large dataset of synthetic coronary stenoses, hence focusing on epicardial lesions, and evaluating them through high‐fidelity 3D computational fluid dynamics (CFD), we derived reference pressure drops across a diverse range of lesion geometries and flow regimes. These CFD‐derived pressures and flows, along with their corresponding shape coefficients, were used to train a lumped parameter model capable of rapidly estimating trans‐lesional pressure drops. Remarkably, only five shape modes were necessary to effectively describe the geometric variability, underscoring the efficiency of the approach. Compared to a conventional lumped model, our approach significantly improved pressure drop prediction accuracy, especially in the case of irregular stenosis morphologies. Integration of the new data‐driven lumped stenosis model within a 1D pulse wave propagation framework was also successful, aligning simulated pressure and flow waveforms much closer with high‐fidelity CFD‐coupled results. In turn, the estimation of the fractional flow reserve, a clinically validated index of lesion‐specific ischemia, also improved by 18% compared to a conventional lumped model. Although only validated using synthetic lesion data, the model's architecture allows easy integration of additional shape features and lesion‐specific parameters, paving the way for future validation on patient‐derived geometries.

## Graphical abstract

A data‐driven workflow for constructing a geometry‐aware lumped arterial stenosis model: Statistical shape modeling extracts geometry‐specific shape modes from synthetically generated coronary lesion geometries. Large‐scale 3D CFD simulations over varying Reynolds numbers yield the geometries their viscous (Kv) and inertial (Kt) loss coefficients. Then, non‐linear regression models map the extracted shape modes to their corresponding Kv and Kt to produce the final geometry‐specific lumped stenosis model.

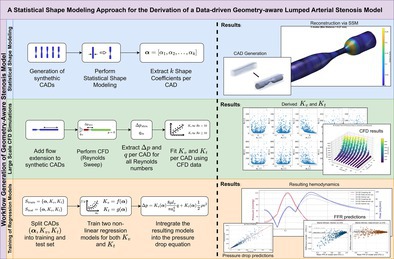

## 1 Introduction

Insight into coronary artery hemodynamics is essential for understanding myocardial perfusion and evaluating epicardial and microvascular disease [1]. Consequently, computational models provide powerful tools for simulating blood flow and pressure in the coronary circulation. Among these, one‐dimensional (1D) models are widely used due to their computational efficiency, enabling the simulation of pulse wave propagation and pressure–flow relationships across arterial networks [2, 3].

A key challenge for these simulations, however, arises in modeling coronary stenotic lesions. The assumptions underlying 1D models, particularly the assumption of minimal radial variations along the vessel wall, limit their ability to capture the pressure losses induced by localized flow disturbances at stenoses [2]. To address this, zero‐dimensional (0D) lumped models are commonly embedded within 1D frameworks to account for the additional resistance and inertial effects across stenotic regions [4, 5, 6]. These lumped models, which draw an analogy to electrical circuits, efficiently capture key hemodynamic features and have shown promise in estimating trans‐lesional pressure drops and predicting fractional flow reserve (FFR), which is a clinically validated index of lesion‐specific ischemia, estimated by the ratio of distal to proximal pressure across a stenosis during conditions of maximal hyperemia [7].

Currently, various lumped models exist that describe the pressure drop across a coronary stenotic lesion. However, most lumped arterial stenosis models assume that the stenosis geometry has a cosinusoidal or trapezoidal shape and/or consider solely the minimal radius of the lesion and that of the healthy vessel [5, 6, 8, 9, 10]. In reality, coronary stenoses have more complex morphologies, ranging from focal to diffuse lesions, and exhibit concentric, eccentric, and irregular reductions in radius, making accurate stenosis modeling with lumped elements challenging [11]. The pressure–flow relationships of the traditional lumped models are typically derived from simplified idealized geometries, which do not adequately capture the complex flow disturbances, such as separation and recirculation, found in real stenoses. Consequently, the underlying simplifications may introduce substantial inaccuracies in both pressure drop and hence FFR estimations, potentially reducing the reliability of these predictions when using these 0D models in clinical settings.

To overcome the limitations of existing lumped models in handling complex lesion morphologies, we propose a geometry‐informed 0D stenosis element that estimates pressure drops based on realistic shape features. This approach utilizes statistical shape modeling (SSM) to describe the stenosis geometry using a compact set of shape coefficients [12, 13]. A large dataset of synthetic coronary stenoses will be generated and analyzed using high‐fidelity 3D CFD simulations to establish reference pressure drops across varying geometries. These shape coefficients, along with their corresponding CFD‐derived pressure and flow data, will form the training set for a lumped parameter model that enables rapid prediction of trans‐lesional pressure drop from shape coefficients and flow alone. In addition to estimating pressure drops, the model will support lesion‐specific FFR prediction, enhancing its potential for clinical integration.

## 2 Methods

To develop a geometry‐aware lumped 0D arterial stenosis model, we established a computational pipeline that links anatomical shape features to hemodynamic outcomes. The basis for the new geometry‐aware lumped arterial stenosis model is the following geometry‐aware instantaneous pressure drop equation:

(1) $$\Delta p=K_{v}\left(\frac{8\;\mu \;l_{s}}{\pi \;a_{0}^{4}}\;q\right)+K_{t}\left(\frac{1}{2}\;\rho \;{\left(\frac{q}{\pi a_{0}^{2}}\right)}^{2}\right).$$

Here, q is the volumetric blood flow, ρ is the density of blood, μ is the dynamic viscosity, $l_{s}$ is the length of the stenotic lesion, $a_{0}$ is the reference radius of a vessel, and $K_{v}$ and $K_{t}$ are geometry‐dependent loss coefficients. Although our pressure drop equation is conceptually comparable to prior studies [5, 10], we will estimate the coefficients $K_{v}$ and $K_{t}$ using a different method: The ultimate goal is to derive the geometry‐dependent $K_{v}$ and $K_{t}$ as functions of statistical shape coefficients derived from a statistical shape model (SSM).

**FIGURE 1:**
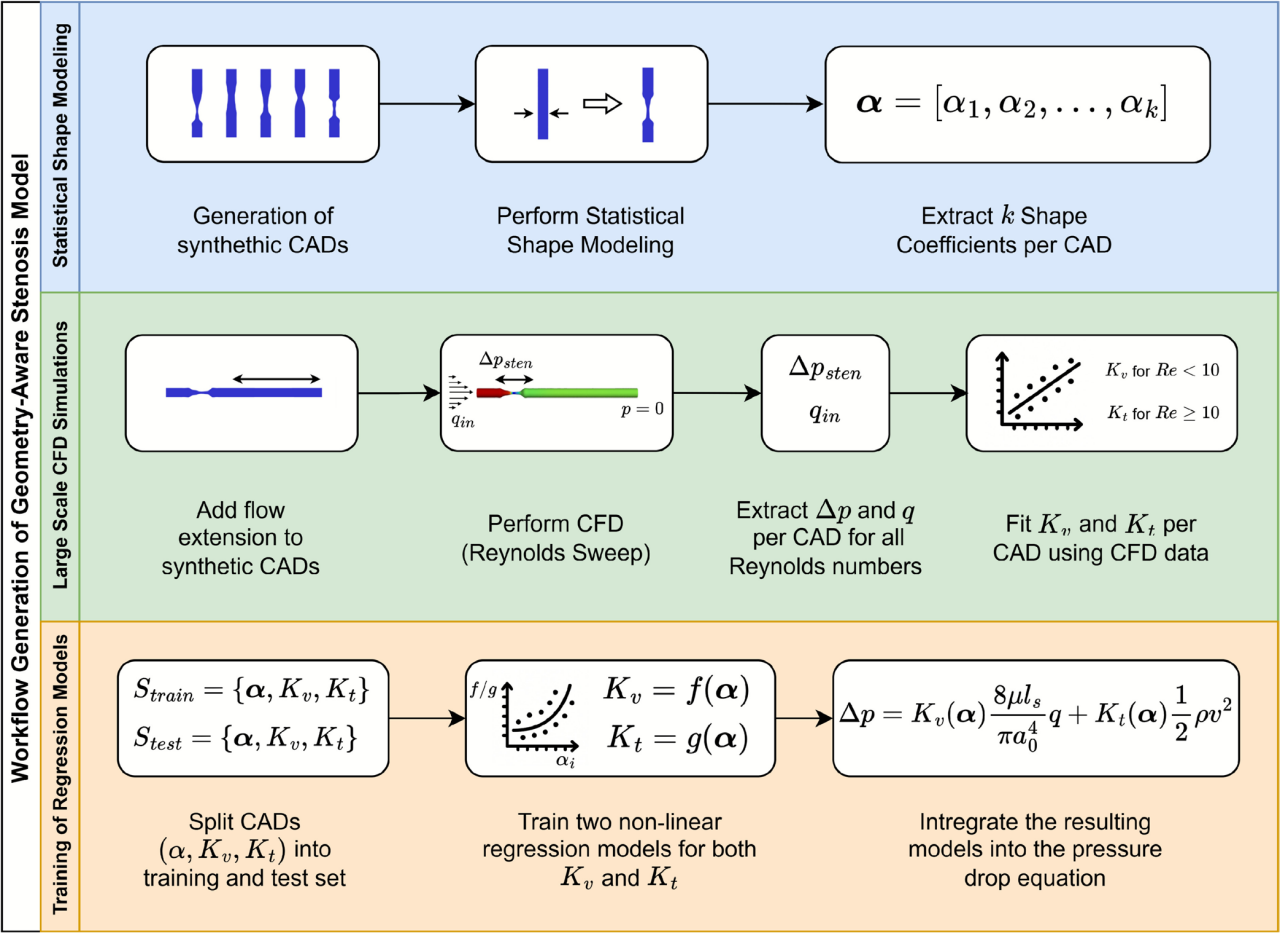
Schematic overview of the overall approach. Here, CAD stands for computer‐aided design; CFD for computational fluid dynamics.

To this end, we follow the methodology depicted in Figure 1. An extensive set of synthetic coronary artery geometries with varying stenosis patterns was generated to better represent realistic morphological variability (blue section in Figure 1). Some idealizations were retained for practicality (e.g., axisymmetry and smooth focal shapes), but the main objective was to move beyond overly simplified geometric shapes and incorporate more physiologically meaningful diameter transitions. Subsequently, SSM is performed on these geometries to derive a finite set of shape coefficients that can easily capture geometric variability and that allows for proper parameterization of each geometry. Second, large‐scale high‐fidelity three‐dimensional (3D) computational fluid dynamics (CFD) simulations were conducted for each geometry to compute pressure drops for varying inflows (green section in Figure 1). Afterwards, the geometry‐specific loss coefficients $K_{v}$ and $K_{t}$ were derived by fitting Equation (1) on the simulation data. Lastly, the resulting dataset, consisting of shape coefficients α, $K_{v}$ and $K_{t}$, was used to train two nonlinear regression models capable of predicting $K_{v}$ and $K_{t}$ based on the geometry‐specific shape coefficients alone (orange section in Figure 1). These regression models were eventually integrated into the pressure drop equation of Equation (1), resulting in the new data‐driven geometry‐aware arterial stenosis model.

### 2.1 Automated Generation of Stenotic Vessel Geometries

**FIGURE 2:**
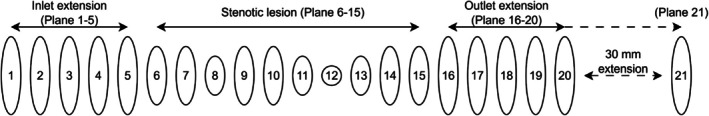
Schematic overview of a typical example of the generated circles on top of the cross‐sectional planes. The planes are indicated by their respective numbers within the circles. The dotted arrows point toward the additional circle on Plane 21, which serves to add an extension to the resulting geometry.

We first need to generate a large set of irregular stenotic lesions to create the training and test sets required to find our new geometry‐aware lumped stenosis element. In this study, as a proof‐of‐principle, we generated synthetic stenotic lesion geometries that go beyond the conventional, idealized, cosinusoidal and trapezoidal profiles by introducing a wider range of shapes, making them more realistic.

solidworks 2024 (Dassault Systèmes, Waltham, MA, USA) was used to generate three‐dimensional geometries of vessels with stenotic lesions. Each geometry is constructed from a series of 20 cross‐sectional equidistant planes (1 mm separation) along the longitudinal axis of the to‐be‐generated vessel. To perform CFD on these geometries, we appended a cylindrical flow extension to the distal end of each domain by adding one extra planar face. The (additional) extension has a constant radius of 1.5mm (the vessel radius) and a length of 10 times the reference diameter (10×3mm=30mm), ensuring that recirculation zones downstream of the lesion are fully captured [14]. This resulted in a total outlet extension of 35 mm. For the inlet, an inlet extension of 5 mm was deemed sufficient, as fully developed flow was prescribed (see Section 2.3.2). A schematic representation for one example geometry can be seen in Figure 2.

Now that we explained the creation of the cross‐sectional planes, we will focus on the creation of the actual geometry. Specifically, how the lesion generation is performed, how we define the stenosis severity and how we introduced inter‐lesion variability between the to‐be‐generated geometries. We define stenosis severity $S_{\mathrm{sev}}=1-\frac{a_{s}}{a_{0}}$, with $a_{s}$ is the minimal lesion radius and $a_{0}$ the reference vessel radius chosen to be 1.5 mm (so $S_{\mathrm{sev}}\in \left[0,1\right]$). To ensure that the severities of the generated geometries are uniformly distributed, we draw $S_{\mathrm{sev}}∼U\left[0.3,0.8\right]$ by using a low‐discrepancy Sobol sequence of 1024 samples, corresponding to stenosis severities of 30% and 80%, respectively. We restrict our sampling to the 30%–80% stenosis interval because below 30% stenting is seldom indicated and above 80% revascularization is almost always required, leaving only this window where the decision remains uncertain. This was generated in Python 2.10 using the Sobol sequence generator in SciPy's quasi‐Monte Carlo module [15].

On the 20, or 21 for CFD, axial planes of Figure 2, we begin by generating circles with the reference radius $a_{0}=1.5\;\mathrm{mm}$ on all planes (i=1,2,…,20/21). To introduce a single focal stenosis within the central lesion segment (i=6,7,…,15), we first sample one minimum lesion severity $S_{\mathrm{sev}}$ from the sample set and set the minimum lesion radius $a_{s}=\left(1-S_{\mathrm{sev}}\right)a_{0}$. We then randomly select the initial stenosis boundary planes at random so that $s_{\text{sten}}\in \left\{6,7,\ldots ,10\right\}$ and $e_{\text{sten}}\in \left\{11,12,\ldots ,15\right\}$. The minimum lesion severity plane $c_{\text{sten}}$ is then computed via the following equation:

(2) $$c_{\text{sten}}=s_{\text{sten}}+2+T\left(\beta \left(e_{\text{sten}}-s_{\text{sten}}-3\right)\right)\;\text{with}\;\beta ∼U\left[0,1\right].$$

Here we have

(3) $$T\left(x\right)=\left\{\begin{array}{cc} \left\lfloor x\right\rfloor & \text{if}\;x\geq 0\\ \left\lceil x\right\rceil & \text{if}\;x<0, \end{array}\right.$$

which truncates x to ensure that the minimum lesion severity plane is an integer. Note that when the initial stenotic region spans only two planes (which must be Plane 10 and Plane 11), the placement of the minimum lesion severity can fall on Plane 12, which lies outside the initially defined lesion region, thus expanding it. We accept this edge case because it still adds realistic variability to our synthetic lesion geometry dataset.

We then assign the reduction profile $a\left(i\right)$ for each plane i by
(4)

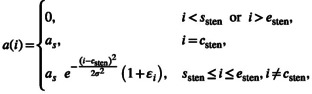

where $s_{\text{sten}}$ and $e_{\text{sten}}$ are still the same initially defined boundary planes, $\sigma ∼U\left[1,3\right]$ controls the Gaussian taper (from sharp when σ≈1 to gradual when σ≈3) and each $\varepsilon_{i}$ introduces an additional ±10% variation. Note that this variation is not applied at the plane $c_{\text{sten}}$, which always receives exactly the target reduction, even in cases where $s_{\text{sten}}=c_{\text{sten}}$ or $e_{\text{sten}}=c_{\text{sten}}$.

**FIGURE 3:**
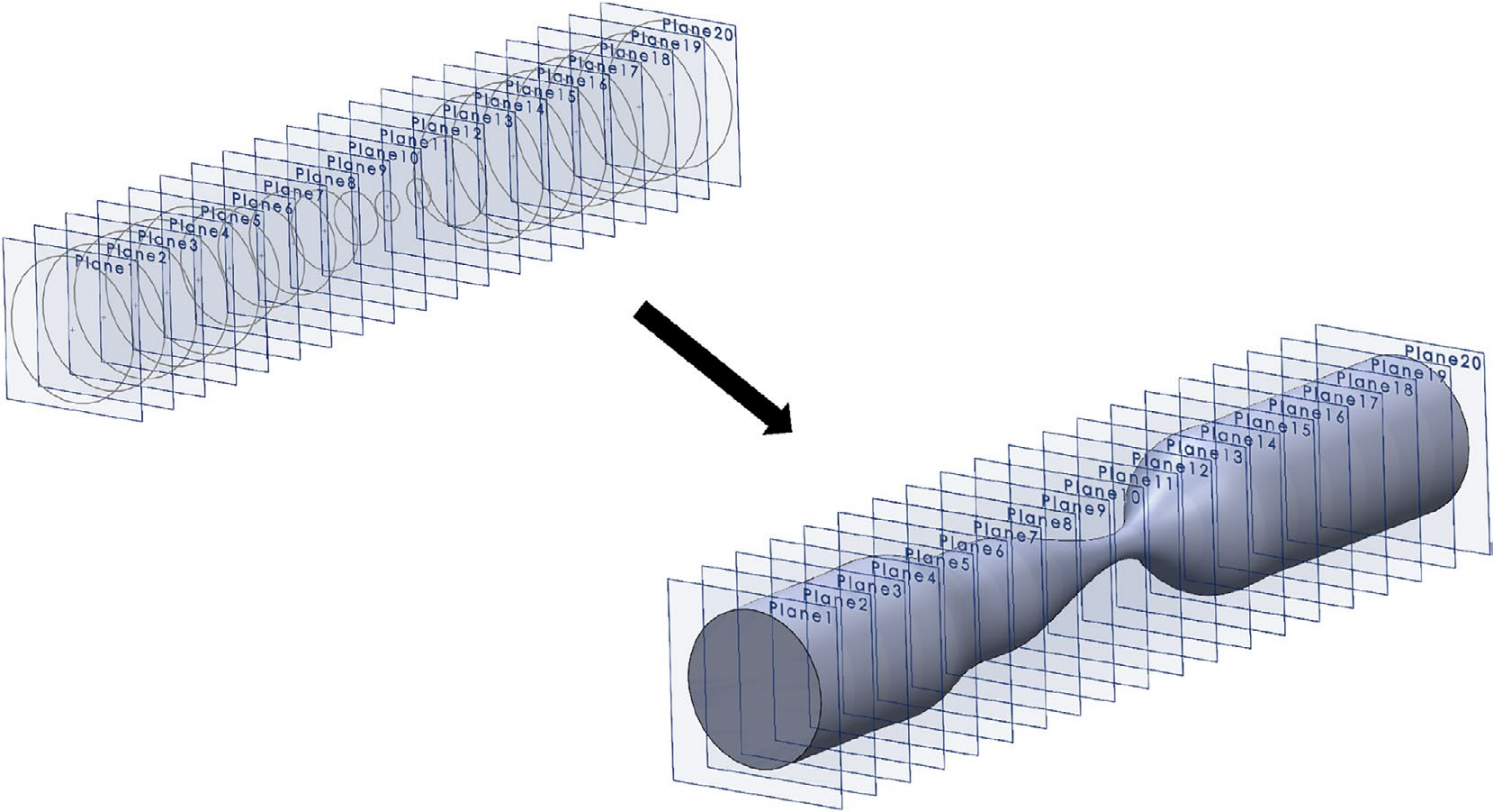
The process of lofting the 20 cross‐sectional planes into a continuous 3D geometry representing a stenotic lesion. Please note that in order to add the extension for CFD, an additional tube is lofted between Planes 20 and 21.

Finally, as can be seen in Figure 3, the series of 20, or 21, cross‐sectional planes is lofted into a continuous three‐dimensional geometry, resulting in a vessel with a distinct, smoothly varying stenotic lesion. Although we maintained axisymmetry around the centerline to simplify the generation process, the resulting geometries still exhibited substantial inter‐geometry variation, even when geometries shared the same severity. To guarantee that the synthetic geometries generated with and without extensions were identical, all randomization was performed pseudorandomly using a fixed seed.

For SSM purposes, we also needed a template geometry (see next section) and to this end we also created one geometry that was generated from lofting Plane 1–20 with all circles with a radius of 1.5 mm, essentially creating a straight tube of 20 mm and a radius of 1.5 mm.

### 2.2 Statistical Shape Modeling

To derive the geometry‐specific shape coefficients, we perform SSM. For the SSM approach, we used the geometries obtained from Section 2.1 that were generated but without the 30 mm extension required for the CFD simulations (so only Plane 1–20). To obtain a parameterized representation of these generated CAD geometries, we adopted an SSM method similar to that employed by Verstraeten et al. [13]. Unlike Verstraeten et al., we use the same straight tube template for every lesion registration rather than deriving a new, optimized cohort mean template.

First, we ensured the generated synthetic stenotic lesion geometries were properly aligned with the template geometry to ensure accurate registration from the template to the target geometry. Alignment was performed in MATLAB R2023a via a two‐step translation: the template geometry's inlet face was moved to *Z* = 0 by subtracting its minimum *Z*‐coordinate, and the inlet centroid (mean *X* and *Y* of points at *Z* = 0) was recorded. Each stenotic geometry was then shifted in *Z* and translated in *X*–*Y* to match the template centroid.

#### 2.2.1 Template Registration

We register the fixed, idealized straight tube template T to each synthetic stenotic lesion $S_{i}$ using the large deformation diffeomorphic metric mapping (LDDMM) framework in Deformetrica [16]. For a detailed explanation of how the template was deformed toward the target geometries we refer you to [13, 16].

Similarly to Verstraeten et al. [13], we optimized the two kernel widths $\lambda_{v}$ and $\lambda_{w}$. The parameter $\lambda_{v}$, which controls the “stiffness” (i.e., smoothness) of the LDDMM deformation, and the parameter $\lambda_{w}$, which sets the scale of the Varifold metric used to compare the deformed template and target. We performed a grid search over $\left(\lambda_{v},\lambda_{w}\right)\in \left[0.1,3\right]\;\mathrm{mm}\times \left[0.1,3\right]\;\mathrm{mm}$, and selected $\lambda_{v}=1\;\mathrm{mm}$ and $\lambda_{w}=2\;\mathrm{mm}$ as they minimized the maximum surface distance between the target geometry and the deformed template geometry, resulting in a maximum distance of only 0.1mm.

#### 2.2.2 Dimensionality Reduction via PCA

After reconstructing all $N_{\mathrm{su}}$ lesion deformations, we collected the initial momenta $p_{i}^{*}$, for each geometry. The *x*, *y* and *z*‐component of $p_{i}^{*}$ were then cast into a single $3N_{v}$‐dimensional row vector using the operator V, forming the momentum vector:
(5)

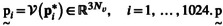




These vectors were then stacked into a data matrix:
(6)

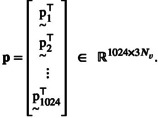




Principal component analysis (PCA) was performed to reduce the dimensionality of the data matrix p using MATLAB 2023a [17]. The prinicpal components, or shape modes, resulting from the PCA describe in which directions the data varies the most. Each set of momenta is therefore approximated by the mean set of momenta plus a linear combination of these PCA‐derived shape modes:
(7)

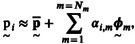

where $N_{m}$ is the total number of shape modes, 
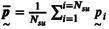
 is the mean set of momenta (sample mean), 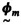 are the PCA shape modes, and $\alpha_{i,m}$ are the corresponding shape coefficients for the *m*‐th shape mode and the *i*‐th geometry.

Consequently, each geometry can be succinctly characterized by the coefficient vector of the form:

(8) $$\alpha_{i}\;=\;\left[\alpha_{i,1},\alpha_{i,2},\ldots ,\alpha_{i,N_{m}}\right]\;\in \;{\mathbb{R}}^{N_{m}}.$$

The shape coefficient vector was intentionally defined in a generic form, as its dimensionality depends on the number of shape modes selected to capture the desired level of geometric variability. The coefficients are dimensionless and do not carry direct geometric interpretations on an element‐by‐element basis, since their influence depends on the specific statistical shape basis derived from the training set. This flexible formulation allows the framework to accommodate different parameterizations without loss of generality.

### 2.3 Large Scale CFD

Having obtained the lesion‐specific shape coefficients $\alpha_{i}$ for all 1024 lesions, we next performed large‐scale CFD simulations (green section of Figure 1). Our aim was to compute geometry‐specific benchmark pressure‐flow relationships, from which we derive the lesion‐specific pressure‐loss coefficients $K_{v}$ and $K_{t}$ as a function of $\alpha_{i}$, respectively. To this end, we conducted an extensive series (see Section 2.3.3) of CFD simulations to generate ground‐truth pressure drop values and the corresponding flows for each synthetic lesion geometry across a broad spectrum of Reynolds numbers. The Reynolds number (Re) is defined as

(9) Re=ρvavgDμ,

where ρ denotes the fluid density, $v_{\mathrm{avg}}$ the mean inlet velocity, D the vessel diameter at the inlet, and μ the dynamic viscosity.

#### 2.3.1 Volumetric Meshing

The generated synthetic stenotic lesion geometries, including the 30 mm extension, from Section 2.1, were imported into Fluent Meshing 2024R1 (Ansys Inc., Canonsburg, PA, USA) for volumetric meshing. The watertight geometry workflow was used, in which polyhedral cells were chosen to discretize the volume domain. No prism boundary‐layer extrusion was used, and no manual local sizing (edge/face/body controls) was applied. For the surface mesh, the Curvature & Proximity size function was enabled with a global growth rate of 1.2 (each successive element at most 1.2 times larger than its neighbor), and proximity (“gap”) capture was kept at default severity (cells per gap = 1, scoped to edges).

**FIGURE 4:**
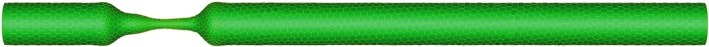
A side view of a typical volumetric mesh resulting from volumetric meshing a CAD geometry.

A detailed mesh convergence analysis had already been performed in our previous study [18] using uniform meshes. In the present work, we therefore adopted an optimized non‐uniform mesh to reduce computational cost while maintaining numerical accuracy. The CAD geometries in this study were meshed with polyhedral cells with a minimal and maximal cell length of 0.05 and 1.25 mm, respectively, resulting in a non‐uniform surface mesh. For the volume mesh, a maximum cell length of 0.10 mm was used with the same growth rate of 1.2. This resulted in a non‐uniform optimized mesh of only 7224 cells. A representative example of such an optimized mesh is shown in Figure 4. The smallest elements are concentrated near the lesion region, where higher spatial resolution is required, while the inlet and outlet extensions feature larger elements to reduce computational cost without compromising accuracy.

**FIGURE 5:**
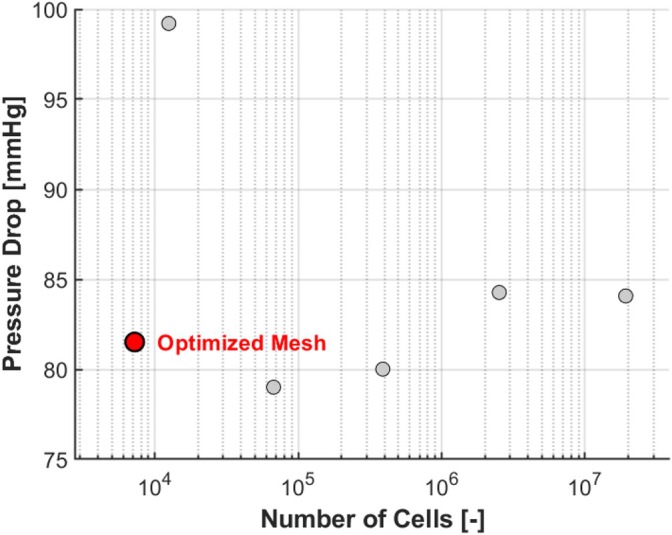
The averaged pressure drop over a typical lesion geometry with $S_{\mathrm{sev}}=0.8$ during the final cardiac cycle for various meshes with different numbers of cells.

We performed an additional mesh analysis (see Figure 5) expanding the mesh analysis of our previous study [19] with an additional simulation, and found that the pressure drop predicted by our optimized mesh of just 7224 cells differed from that of a much finer uniform mesh of 19,210,726 cells by only 2.5 mmHg, which is below the clinically accepted drift threshold of 3 mmHg [20]. For a detailed explanation of the mesh analysis, we refer you to the appendix of our previous study [18], which was done solely with a uniform mesh. The current study adds the optimized mesh (the data point shown in red in Figure 5) since large‐scale CFD simulations required optimized runtimes to ensure practical feasibility. Please note that the smallest number of cells for the optimized mesh does not mean it is the coarsest mesh, as it is not uniform.

Each CAD geometry was meshed once before conducting the CFD simulations, and the resulting mesh was consistently reused for all simulations corresponding to that particular geometry.

#### 2.3.2 Fluid Dynamics Solver

Simulations were performed in ANSYS Fluent 2024 R1 (Ansys Inc.), where the mass and momentum conservation equations are addressed via a finite‐volume method (FVM). For spatial discretization, a second‐order upwind scheme is used. Temporal derivatives are discretized using a second‐order implicit method. The resulting linear system is solved iteratively using the Gauss–Seidel method. A pressure‐based segregated solver employing the SIMPLE algorithm is used, which applies a projection step to enforce continuity before sequentially solving the governing equations.

Laminar blood flow was assumed, with density ρ and dynamic viscosity μ set to 1060 $\mathrm{kg}\cdot m^{-3}$ and 0.0035 $\mathrm{kg}\cdot m^{-1}\cdot s^{-1}$, respectively. The inlet of the 3D domain was subjected to a steady parabolic velocity profile so that the intended Reynolds numbers (Equation 9) are ensured. Reynolds numbers were varied between 0.1 and 500, defined by the inlet velocity using the reference radius of 1.5mm. The upper limit of Re=500 exceeds the mean hyperemic flow rate of 200mL/min observed in [21], which corresponds to Re=428. A zero‐pressure outlet and rigid, no‐slip walls were applied throughout the domain.

For the highest Reynolds number case (Re=500) we estimated a minimum cell size of $\Delta x_{\min}\approx 5\times 10^{-5}\;m$ and a characteristic velocity u≈0.55m/s, yielding an explicit‐CFL time step of

(10) $$\Delta t=\frac{\Delta x_{\min}}{u}\;\approx \;\frac{5.0\times 10^{-5}\;m}{0.55\;m/s}\;\approx \;9.1\times 10^{-5}\;s,$$

which was rounded to $\Delta t=1\times 10^{-4}\;s$. This single time step was then applied to all lower‐Reynolds‐number simulations as well under the assumption that reduced velocities at lower Re would then also maintain CFL<1.

Up to 200 solver iterations were allowed within each physical time step to ensure convergence of the governing equations, which was typically reached well before the iteration limit. The solver could stop the iterations and proceed to the next time step once the globally scaled $L_{1}$‐norm of the continuity and velocity residuals were below $1\times 10^{-4}$ [−] and $1\times 10^{-5}$ [−], respectively. The calculation was advanced in successive Δt increments until the inlet pressure reached a steady state; the simulation was halted when the $L_{1}$‐norm of the relative change in inlet pressure over five consecutive time steps dropped below $1\times 10^{-4}$ [Pa].

#### 2.3.3 Generation of the CFD Data Set

Simulations were run for all 1024 geometries for various Reynolds numbers: [0.1, 1, 10, 50, 100, 150, 200, 250, 300, 350, 400, 450, 500]. The resulting pressure drop and the accompanying flow were stored for each simulation. To isolate the pressure loss solely due to the stenotic lesion, we subtract the baseline Poiseuille pressure drop through the straight, non‐stenotic segments of total length $l_{\mathrm{ext}}=40\;\mathrm{mm}$ (total length of Planes 1–5 and 16–21 in Figure 2) from the total simulated pressure drop between inlet and outlet. The baseline pressure drop is

(11) $$\Delta p_{\text{Pois}}=\frac{8\;\mu \;l_{\mathrm{ext}}\;q_{\text{in}}}{\pi \;a_{0}^{4}},$$

where $q_{\text{in}}$ the imposed volumetric flow rate, and $a_{0}$ the radius of the extensions. Since the outlet pressure is zero ($p_{\text{out}}=0\;\text{mmHg}$), the area‐averaged inlet pressure ${\bar{p}}_{\text{in}}$ equals the total simulated drop. Hence, the stenotic pressure drop in the 3D model becomes

(12) $$\Delta p_{\text{sten},3D}={\bar{p}}_{\text{in}}-\Delta p_{\text{Pois}}={\bar{p}}_{\text{in}}-\frac{8\;\mu \;l_{\mathrm{ext}}\;q_{\text{in}}}{\pi \;a_{0}^{4}}.$$

Simulations yielding a trans‐stenotic pressure drop above 60mmHg were excluded to maintain physiological relevance. In the IDEAL study [22], severe stenoses (FFR ≤0.50) exhibit a mean whole‐cycle pressure drop of 54.6mmHg, while intermediate lesions around FFR ≈0.80 in that study produced mean drops of 13.0mmHg and 22.2mmHg. Because FFR assessment is most critical near the clinical decision threshold of 0.80, where pressure drops are typically in the 15−25mmHg range [22], a 60mmHg cutoff lies well above the physiologically relevant values, excluding only the most extreme outliers.

#### 2.3.4 Estimation of the Loss Coefficients $K_{v}$ and $K_{t}$

The resulting CFD outputs (pressure drop $\Delta p_{\text{sten},3D}$ and inlet flow rate q) are used to estimate loss coefficients $K_{v}$ and $K_{t}$ for each geometry. First, per Reynolds simulation and per geometry, the inlet flow rate q is used to derive the terms:

(13) $$h_{1}\left(q\right)=\frac{8\;\mu \;l_{s}}{\pi \;a_{0}^{4}}\;q,\;\text{and}\;h_{2}\left(q^{2}\right)=\frac{1}{2}\;\rho {\left(\frac{q}{\pi \;a_{0}^{2}}\right)}^{2}.$$

Similar to Heinen et al. [10], the data is split per geometry at Re=10. For the low‐Reynolds (Re<10) regime, we can ignore the quadratic *h*_2_‐term and only focus on the linear effect contributing to the pressure drop, resulting in

(14) $$\Delta p=K_{v}\;h_{1}\left(q\right),$$

where $h_{1}\left(q\right)$ and Δp are known from the simulations and $K_{v}$ is obtained by an ordinary least‐squares fit of Δp versus $h_{1}\left(q\right)$ through the origin, minimizing the sum of squared residuals. In the high‐Reynolds regime (Re≥10), both linear and non‐linear effects contribute to the pressure drop. By subtracting the linear term,

$$\Delta p-K_{v}\;h_{1}\left(q\right)=K_{t}\;h_{2}\left(q^{2}\right),$$

and using the previously determined value of $K_{v}$, we isolate and determine the loss coefficient $K_{t}$. Here $h_{2}\left(q^{2}\right)$ is known, and $K_{t}$ is obtained by an ordinary least‐squares fit of the residual pressure drop $\Delta p-K_{v}\;h_{1}\left(q\right)$ against $h_{2}\left(q^{2}\right)$, also through the origin. Finally, one value of $K_{v}$ and one value of $K_{t}$ are extracted per geometry, forming the training set together with the shape coefficients $\left(\alpha_{1},\ldots ,\alpha_{N_{m}}\right)$.

### 2.4 Training of the Data‐Driven Stenosis Model

Once the loss coefficient $K_{v}$, loss coefficient $K_{t}$, and the shape coefficients $\alpha_{i}$ for each geometry were obtained, we are ready to find a geometry‐dependent expression for $K_{v}$ and $K_{t}$ by training a nonlinear regression model (blue section in Figure 1). The training set consists of the data of 90% of the geometries, whereas the data of the remaining 10% of the geometries is put aside as test data. The exact number of geometries in the training and test set is shown in Table 1.

**TABLE 1:** Training and test set split.

Set	Number of geometries
Training	918
Test	103

*Note:* 1021/1024 simulations converged.

#### 2.4.1 Training of the Regression Models for $K_{v}\left(\alpha \right)$ and $K_{t}\left(\alpha \right)$

The aim is to predict the loss coefficients, $K_{v}$ and $K_{t}$, from $N_{u}$ shape coefficients $\alpha =\left[\alpha_{1},\ldots ,\alpha_{N_{u}}\right]$, where $N_{u}\leq N_{m}$. To this end, we employed two Gaussian process regression (GPR) models for predicting $K_{v}$ and $K_{t}$ respectively, as they provide a nonparametric regression framework with high flexibility in capturing complex data relationships [23]. Moreover, Gaussian processes naturally yield a full posterior predictive distribution, so that for each new input, we obtain both a mean prediction and an uncertainty estimate (the predictive variance). The GPR uses a composite kernel:

(15) $$k\left(x_{i},x_{j}\right)\;=\;C\;k_{\mathrm{RBF}/\text{Matern}}\left(x_{i},x_{j}\right)\;+\;k_{\text{White}}\left(x_{i},x_{j}\right),$$

where $x_{i},x_{j}\in {\mathbb{R}}^{d}$ are the input feature vectors and C is a constant scaling factor. The first kernel consists of either an RBF kernel ($k_{\mathrm{RBF}}$) or a Matérn kernel ($k_{\text{Matern}}$), both of which can handle nonlinear data and are highly flexible. The Matérn kernel additionally introduces adjustable smoothness, via ν, which allows capturing both smooth and more irregular behaviors without overfitting. The mathematical definitions of the RBF and Matérn kernel are as follows:

(16) $$k_{\mathrm{RBF}}\left(x_{i},x_{j}\right)=e^{\frac{{\left\|x_{i},x_{j}\right\|}_{2}^{2}}{2\ell^{2}}}\;\text{and}$$

(17) $$k_{\text{Matern}}\left(x_{i},x_{j}\right)=\frac{1}{\Gamma \left(\nu \right)\;2^{\nu -1}}{\left(\frac{\sqrt{2\nu }}{\ell }∥x_{i}-x_{j}{∥}_{2}\right)}^{\nu }K_{\nu }\;\left(\frac{\sqrt{2\nu }}{\ell }∥x_{i}-x_{j}{∥}_{2}\right).$$

Here, $∥x_{i}-x_{j}{∥}_{2}$ is the Euclidean distance, $\ell =\left(\ell_{1},\ldots ,\ell_{N_{u}}\right)$ the length‐scale vector (each $\ell_{i}$ initialized to 1 and constrained to [$10^{-3},10^{5}$]), ν the smoothness parameter, $\Gamma \left(\cdot \right)$ the gamma function, and $K_{\nu }\left(\cdot \right)$ the modified Bessel function of the second kind. The second kernel is a White (noise) kernel ($k_{\text{White}}$), which is added to account for numerical errors and residual variability, ensuring realistic uncertainty intervals. The White kernel is given by:

(18) $$k_{\text{White}}\left(x_{i},x_{j}\right)=\left\{\begin{array}{cc} \sigma^{2}, & x_{i}=x_{j},\\ 0, & x_{i}\neq x_{j}. \end{array}\right.$$

Here, $\sigma^{2}$ is the noise‐level hyperparameter representing the variance of the additive white noise.

Optuna was used to find the optimal kernels and other hyperparameters. It is a framework for hyperparameter optimization that uses Bayesian optimization via its TPESampler algorithm, where TPE stands for Tree‐structured Parzen Estimator [24]. In our sampling strategy, a subset of hyperparameters is chosen randomly from the hyperparameter ranges shown in Table 2.

**TABLE 2:** Optuna hyperparameter search space.

Parameter	Sampling	Range/values	Condition
Polynomial degree	Integer	1 to 3	—
Use natural‐log transform	Categorical	{False, True}	—
kernel	Categorical	{Matern, RBF}	—
ν	Categorical	{0.5, 1.5, 2.5}	kernel = Matern
C	Log‐Uniform	$\left[10^{-1},10^{5}\right]$	—
$\sigma^{2}$	Log‐Uniform	$\left[10^{-6},10^{1}\right]$	—

Hyperparameter Polynomial degree determined whether or not enriching the original $\alpha =\left[\alpha_{1},\ldots ,\alpha_{N_{m}}\right]$ with either second‐ or third‐order terms was essential to capture the non‐linear behavior of the loss coefficients. It enables the GPR model to model both quadratic effects and mode‐to‐mode interactions. The hyperparameter Use natural‐log transform could deal with skewness within the data and promote a more Gaussian target distribution [25, 26], which could result in an improved learning outcome. It is applied as a natural log‐transform with a small offset:

(19) $$y_{v}=\ln\left(K_{v}+\varepsilon \right),\;y_{t}=\ln\left(K_{t}+\varepsilon \right),$$

where $\varepsilon =10^{-6}$ ensures the argument of the logarithm is strictly positive as $K_{v},K_{t}>0$.

In each iteration, Optuna first explores the search space by sampling hyperparameter configurations at random and evaluating their performance according to a specified objective metric. For each individual hyperparameter x, it then splits the trials into two sets: the best‐performing values of x are used to fit a Gaussian mixture model (GMM) $l\left(x\right)$, while the remaining values define a second GMM $g\left(x\right)$ [24]. New candidate values for x are proposed by selecting the option that maximizes the ratio $l\left(x\right)/g\left(x\right)$. This cycle continues until the preset number of trials is reached. For a robust selection of optimal hyperparameters, k‐fold cross validation (k=5) was employed within this search strategy over 20 optimization rounds, evaluating each trial by minimizing the mean squared error (MSE), which is given by:

(20) $$\mathrm{MSE}\left(y,\hat{y}\right)\;=\;\frac{1}{n}\sum_{i=1}^{n}{\left(y_{i}-{\hat{y}}_{i}\right)}^{2}.$$

Here, $y_{i}$ is the true target for the i‐th sample, ${\hat{y}}_{i}$ is the predicted value for the i‐th sample, n is the total number of samples. Lastly, we used a fixed random seed to ensure reproducible optimization of the kernel parameters.

#### 2.4.2 Model Evaluation

The performance of the nonlinear regression models for $K_{v}$ and $K_{t}$ is evaluated on the held‐out geometries in the test set. We quantify predictive performance by computing the coefficient of determination of the linear regression line ($R_{y=x}^{2}$) between the predicted and true loss coefficients, the root mean squared error (RMSE) of the predicted loss coefficients, and the mean predictive standard deviation σ of the loss coefficients.

Next, we evaluate the new geometry‐aware lumped arterial stenosis model, that is, the data‐driven pressure drop equation by deploying both GPR models for $K_{v}$ and $K_{t}$. For each geometry i in the test set, we predict $K_{v,i}$ and $K_{t,i}$ from its shape‐mode vector $\alpha_{i}$ using the GPR models, and then compute the estimated pressure drop:

(21) $$\Delta p_{\text{pred}}=K_{v,i}h_{1}\left(q\right)+K_{t,i}h_{2}\left(q^{2}\right).$$

Prediction quality is then assessed by comparing $\Delta p_{\text{pred}}$ to the CFD simulated pressure drop $\Delta p_{\mathrm{CFD}}$ via the same metrics ($R_{y=x}^{2}$, RMSE, and σ) as for the nonlinear $K_{v}$ and $K_{t}$ regression models.

### 2.5 Data‐Driven Stenosis Model Performance Comparison

To assess the added benefit of our new data‐driven geometry‐aware lumped stenosis element, we compare the performance with the lumped stenosis model of Heinen et al. [10]. The geometry‐based model of Heinen et al. relates pressure and flow over iliac artery stenoses based on typical dimensionless stenosis characteristics, which makes it likely applicable to coronary lesions (we refer the reader to the appendix of [19] for a CFD‐based verification). We selected this model because it was shown that it could more accurately predict the in vivo pressure drop over iliac artery stenoses than other lumped models that were not using severity‐dependent loss coefficients [10]. However, the model of Heinen et al. does assume a cosinusoidal lesion shape. The lumped stenosis model of Heinen et al. is of the form:

(22) $$\Delta p=K_{v}\frac{8\mu l_{s}}{\pi a_{0}^{4}}\;q\;+\;K_{t}\frac{\rho }{2\pi^{2}a_{0}^{4}}{\left[{\left(\frac{a_{0}}{a_{s}}\right)}^{2}-1\right]}^{2}q^{2}.$$

Here, μ is the dynamic viscosity, $l_{s}$ is the length of the stenotic lesion, $a_{0}$ is the radius of a non‐stenotic part of the vessel, $a_{s}$ is the minimal stenosis radius, and $K_{v}$ and $K_{t}$ are geometry‐dependent loss coefficients. Unlike our SSM‐based geometric dependency, Heinen et al. derive the loss coefficients solely from the following geometrical features: the lesion's minimal and reference areas and its length. We refer the reader to Heinen et al. [10] for a more detailed description of the original model. The constant parameters used to evaluate the Heinen model in this study can be found in Table 3.

**TABLE 3:** Heinen's geometry‐based lumped stenosis model constant parameters.

Symbol	Value
μ	0.0035 Pa s
ρ	1060 kg m^−3^
$a_{0}$	1.5 mm
$l_{s}$	10 mm

#### 2.5.1 Hemodynamic Comparison

Using three heuristically‐chosen geometries that were included in the test set of Table 1, we evaluated our newly trained geometry‐aware stenosis model, in its intended setting, by deploying it within our 1D pulse wave propagation model (as detailed in [18]). At each time step, the instantaneous 1D flow $Q_{1D}$ obtained from the 1D element directly proximal to the lumped stenosis element is used to update the resistance of the lumped stenosis element according to the relation

(23) $$R\left(Q\right)=K_{v}\;\frac{8\;\mu \;l_{s}}{\pi \;a_{0}^{4}}\;+\;K_{t}\;\frac{\rho }{2\pi^{2}a_{0}^{4}}\;{\left|Q_{1D}\right|}_{\mathrm{sm}},$$

where ${\left|Q_{1D}\right|}_{\mathrm{sm}}$ is the flow magnitude after an under‐relaxation step. We perform under‐relaxation with smoothing factor α=0.3 (i.e., 30% of the previous smoothed value and 70% of the current raw magnitude) to damp sudden changes and prevent numerical instabilities during near‐zero and reversed systolic flow:

(24) $${\left|Q_{1D}\right|}_{\mathrm{sm}}^{n}=\alpha \;{\left|Q_{1D}\right|}_{\mathrm{sm}}^{n-1}\;+\;\left(1-\alpha \right)\;|Q_{1D}^{n}|.$$

The resulting pressure drop is then simply $\Delta P=R\left(Q\right)\;Q$. In parallel, a full 3D stenosis geometry was coupled to the same 1D framework via a steady 1D–3D interface approach at the lesion location (as described in [18]), ensuring identical inflow conditions for both models. For each test case, we compared pressure and flow waveforms resulting from the implementation of the two lumped stenosis elements—the Heinen et al. model and our geometry‐aware data‐driven model—within the 1D pulse wave propagation model with the pressure and flow waveforms resulting from the steady‐coupled 3D CFD lesion simulations embedded in the 1D pulse wave propagation model. In these comparisons, pressure waveforms were extracted just downstream of the stenosis in the 1D pulse wave model, while flow waveforms were taken immediately upstream of the lesion.

#### 2.5.2 FFR Prediction Comparison

Afterwards, using $\Delta p_{\mathrm{CFD}}$ and $\alpha_{i}$ for all geometries that were included in the test set of Table 1, we evaluated our newly trained geometry‐aware stenosis model using the FFR to showcase the future potential for patient‐specific and non‐invasive predictions. The FFR is estimated by the ratio of the mean coronary pressure distal to a stenosis, $P_{\text{distal}}$, to the mean aortic pressure, $P_{\text{aorta}}$, under conditions of maximal hyperemia [7]. For each geometry and their respective Reynolds simulations in the test set, we compute the resulting pressure drops $\Delta p_{\text{data}-\text{driven}}\;\text{and}\;\Delta p_{\text{Heinen}}$ from the geometric parameters ($\alpha_{i},l_{s},a_{0},a_{s}$) using our data‐driven model and Heinen et al.'s [10] model, respectively, while the CFD‐based pressure drop $\Delta p_{\mathrm{CFD}}$ is taken directly from the test set simulations. Since we do not have the distal and aortic pressure of our CFD simulations, but just the pressure drop, we compute the FFR for all simulations (data‐driven,Heinen,CFD) directly from that pressure drop via:

(25) $$\mathrm{FFR}=\frac{P_{\text{distal}}}{P_{\text{aorta}}}=\frac{P_{\text{aorta}}-\Delta p}{P_{\text{aorta}}}=1-\frac{\Delta p}{P_{\text{aorta}}}\approx 1-\frac{\Delta p}{100\;\text{mmHg}}.$$

Here we have taken $P_{\text{aorta}}=100\;\text{mmHg}$, the mean of typical systolic (120 mmHg) and diastolic (80 mmHg) pressures [27]. This yields ${\mathrm{FFR}}_{\text{data}‐\text{driven}}$, ${\mathrm{FFR}}_{\text{Heinen}}$ and ${\mathrm{FFR}}_{\mathrm{CFD}}$, all obtained using a fixed flow rate. Agreement between each lumped model and the CFD “gold‐standard” was assessed by numerical accuracy (absolute error ≤0.02 relative to ${\mathrm{FFR}}_{\mathrm{CFD}}$) and by binary classification (FFR≤0.80 vs. >0.80), since that threshold drives clinical decision‐making [7]. Note that this estimated FFR is not the actual FFR, but a surrogate, as we have assumed an aortic pressure of 100 mmHg.

Finally, using the configurations described in Section 2.5.1, we computed FFR by deriving proximal (aortic) and distal pressures from the 1D pulse‐wave propagation model with the stenosis models (3D CFD, data‐driven, and Heinen) in place (similar to [18]). We compared the resulting FFR values from both lumped stenosis models to those obtained via 1D–3D coupling, which serves as a reference. In contrast to the fixed‐flow analysis above, the 1D–3D coupled simulations resolve the full cardiac cycle, and FFR is obtained as the time‐averaged ratio of distal to aortic pressure.

### 2.6 Execution and Infrastructure

To perform the large‐scale CFD simulations required for our study, we implemented a fully automated and parameterized workflow using the PyFluent library, which enabled scalable execution and flexible control. This workflow was deployed on the Ares High‐Performance Computing (HPC) cluster at the Academic Computer Center Cyfronet in Kraków, with compute nodes configured with 48 cores and 192 GB RAM (2× Intel Xeon Platinum 8268 CPUs, 2.90 GHz). Each simulation task was assigned 8 cores and 32 GB RAM, providing a good balance between performance and resource utilization, resulting in 80%–90% SLURM job efficiency.

In total, 1024 geometries were simulated. Each was processed as an independent job submitted via the Slurm array job mechanism. We configured the array job to limit the number of simultaneously running tasks to 50, which ensured that at most 50 simulations ran concurrently. As soon as one task completed, the next pending task in the array was launched. This strategy allowed us to maintain continuous utilization of available computational resources without overloading the Fluent license server.

The simulation workflow for each geometry consisted of two stages: meshing, performed once per geometry, and solver session, during which 13 separate simulations were run in sequence, each corresponding to a different Reynolds number. To minimize overhead, all 13 (for each Reynolds number) simulations were executed within a single Fluent session by resetting the solver setup between runs, avoiding repeated PyFluent initializations and license server connections. The total execution time per geometry, including meshing and all 13 simulations, averaged around 40 min. Across the experiment implementation, tests, and the entire simulation campaign, we consumed approximately 6000 CPU hours.

Simulation results were collected in a structured directory hierarchy, with each geometry producing a CSV file summarizing all simulation outputs. This included metadata such as Reynolds number, inlet pressure and flow, convergence status, and iteration count. Following each full array job execution, the result files were merged to verify completion. Any failed or non‐converged cases could be efficiently resubmitted using a new array job targeting only the affected geometry IDs.

For the verification with 1D‐3D coupled simulations, we employed the Model Execution Environment [28], which facilitated reproducible execution of the 1D‐3D coupling pipelines, model versioning, simulation tracking, as well as browsing execution and result history. We refer the reader to our previous work [18] for more information on the execution and infrastructure of our 1D‐3D coupled simulations.

## 3 Results

### 3.1 Generation of CAD Geometries

**FIGURE 6:**
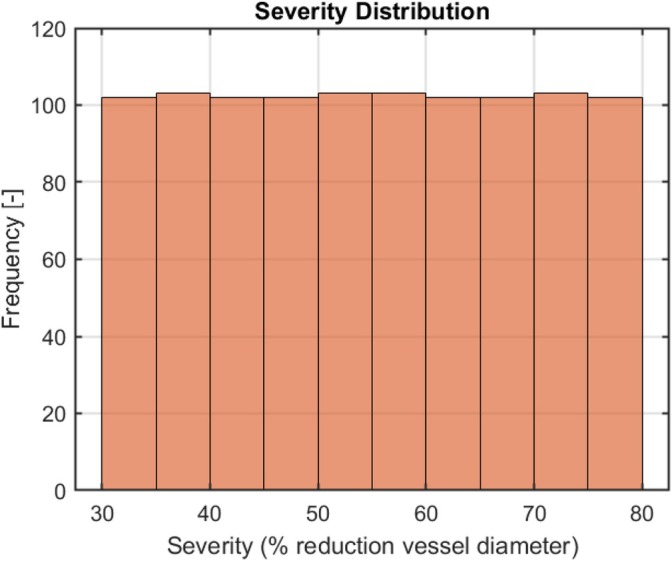
Histogram of severity values for all 1024 geometries generated in SolidWorks CAD, showing the frequency distribution of the computed severity metric across the dataset. Each geometry corresponds to a single severity value (percentage reduction in vessel diameter), uniformly sampled between 30% and 80%.

The CAD geometries representing a stenotic lesion were generated automatically using Sobol low‐discrepancy sampling to ensure a uniform distribution of maximum diameter reduction. As shown in Figure 6, the resulting CAD geometries exhibited a uniform distribution of stenosis severity, with diameter reductions ranging between 30% and 80%.

**FIGURE 7:**
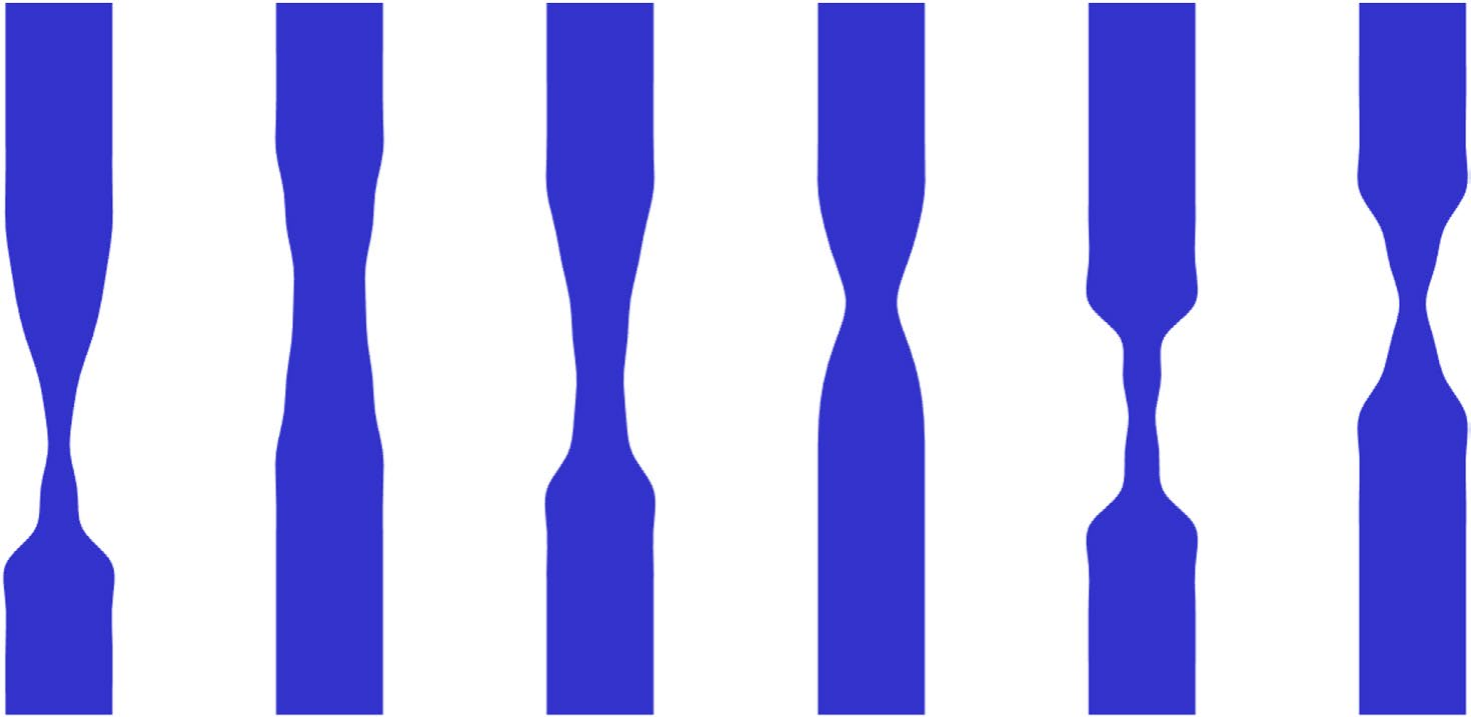
Cross‐sectional views of six representative irregular stenotic lesions. Each geometry is shown in cross‐section due to its axial symmetry around the central longitudinal axis. From left to right, the corresponding stenosis severities are 79%, 32%, 56%, 52%, 75%, and 75%.

The automated process generated 1024 CAD geometries of stenotic lesions, with several typical examples illustrated in Figure 7. As shown in Figure 7, many lesions differ from idealized cosinusoidal or trapezoidal profiles. While a few, such as the second lesions from both the left and the right, still approximate a pure cosine shape, most represent more irregular stenotic lesion shapes. Please note that although the final two CAD geometries (on the right) share identical severity percentages (75%), their shapes differ.

### 3.2 Statistical Shape Modeling

**FIGURE 8:**
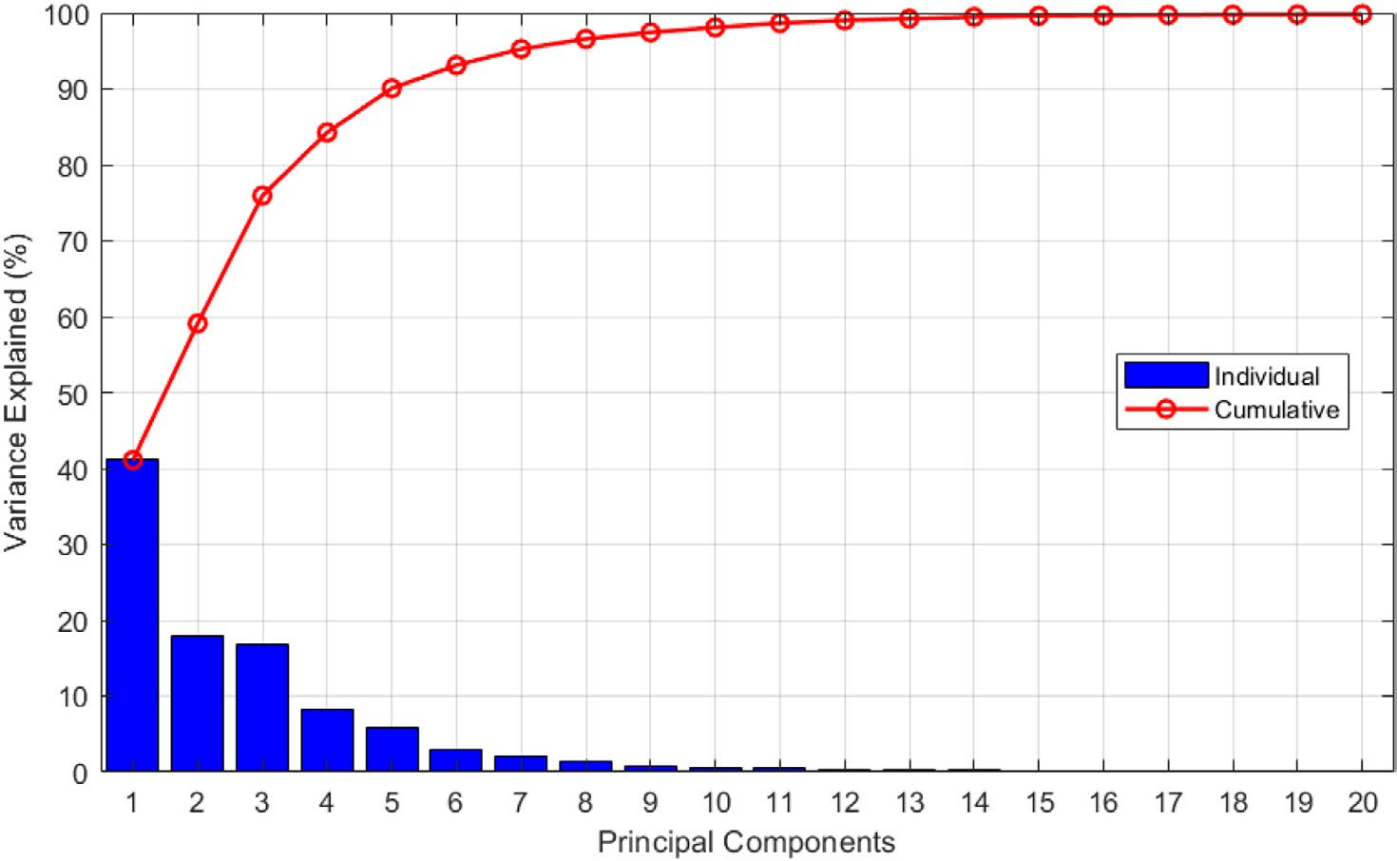
Percentage of variance explained by each of the first 20 principal components (blue bars) and cumulative variance (red line).

Figure 8 shows, as a result of the PCA, the percentage of variance explained by each principal component and the cumulative variance. Based on this PCA, we determined that retaining the first five principal modes ($N_{m}=5$) captures approximately 90% of the total variance. It was initially assumed (and later confirmed) to be sufficient to represent the primary geometric variations and provide sufficient geometric information to predict the lesion‐specific pressure drop accurately.

Consequently, the dimensionality of the lesion geometries has been effectively reduced from $N_{\mathrm{su}}-1=1023$ dimensions to only 5, allowing each geometry to be succinctly characterized by the coefficient vector $\alpha_{i}=\left[\alpha_{i,1},\alpha_{i,2},\alpha_{i,3},\alpha_{i,4},\alpha_{i,5}\right]\in {\mathbb{R}}^{5}$.

**FIGURE 9:**
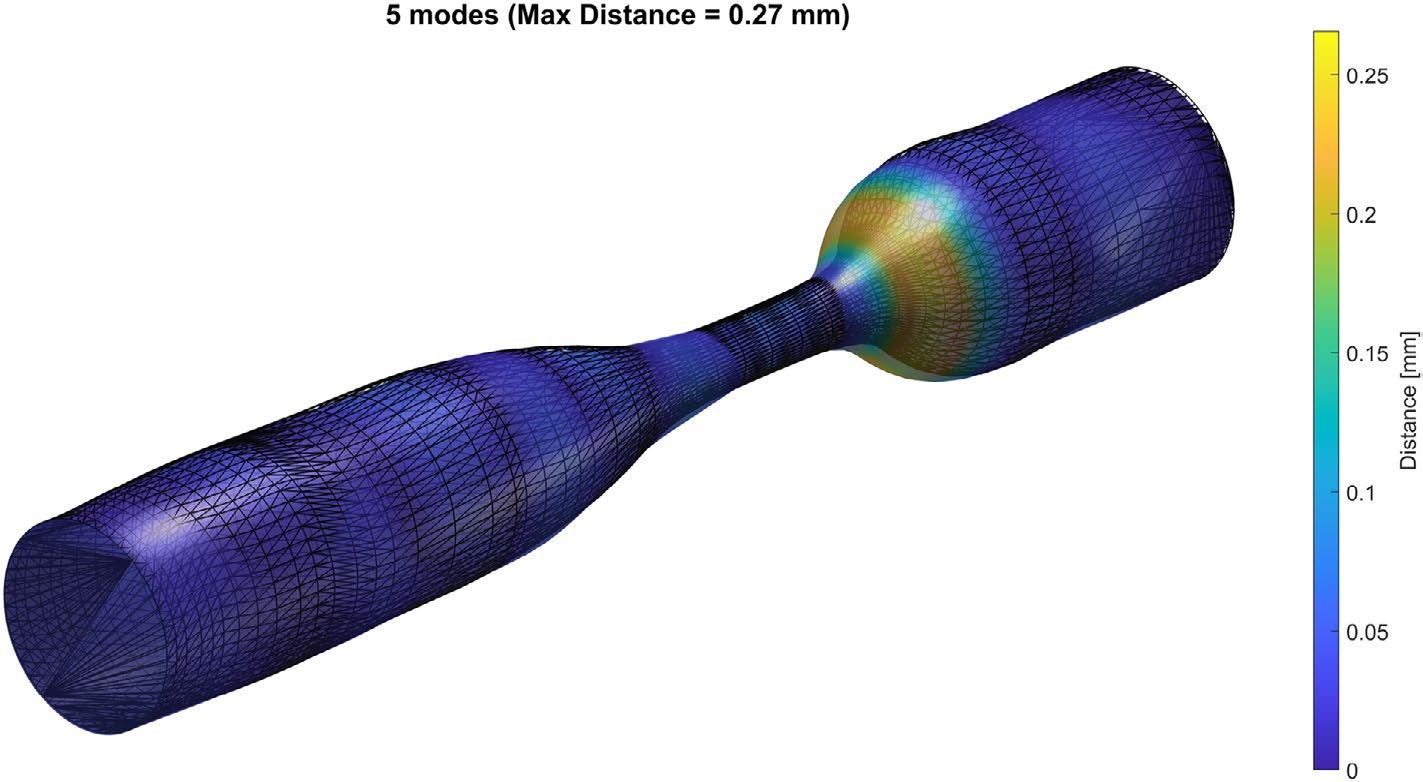
Reconstruction with five shape modes.

**FIGURE A1:**
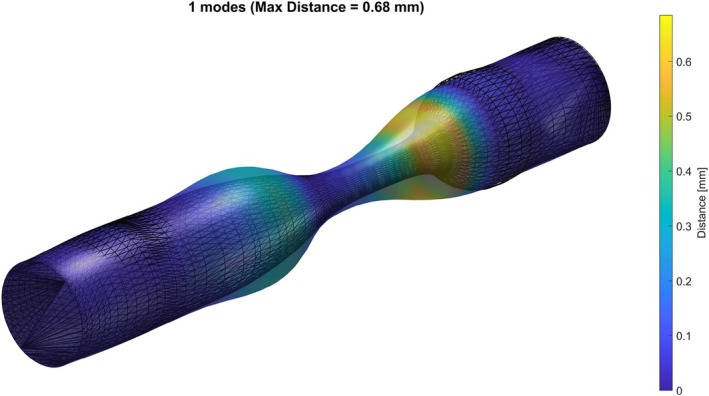
Reconstruction with one shape mode.

**FIGURE A2:**
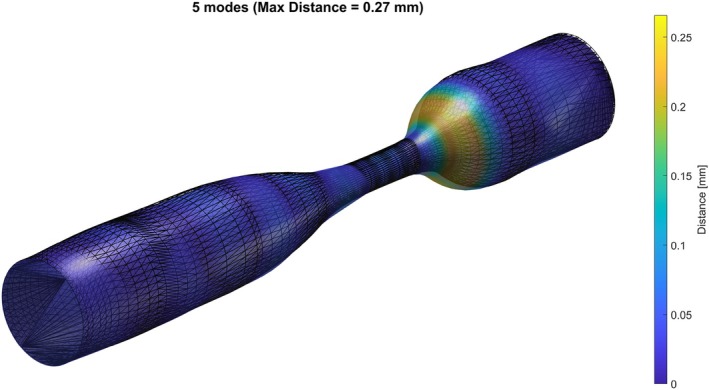
Reconstruction with five shape modes.

**FIGURE A3:**
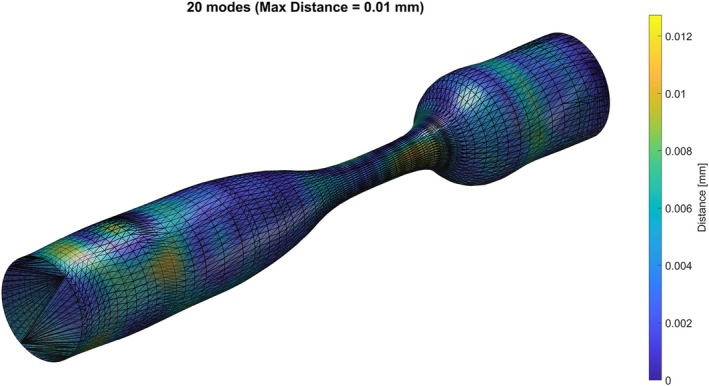
Reconstruction with 20 shape modes.

**FIGURE A4:**
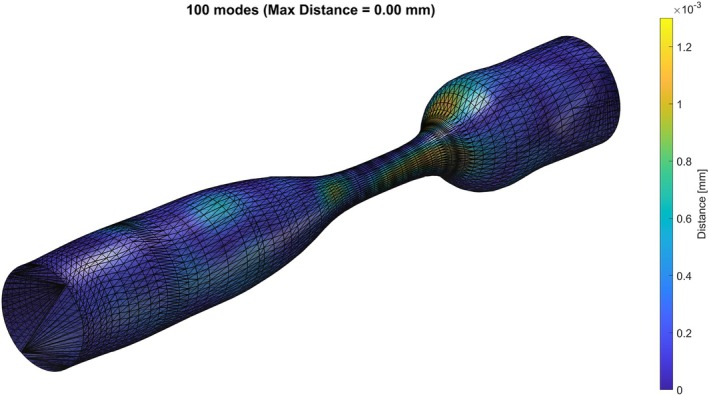
Reconstruction with 100 shape modes.

**FIGURE A5:**
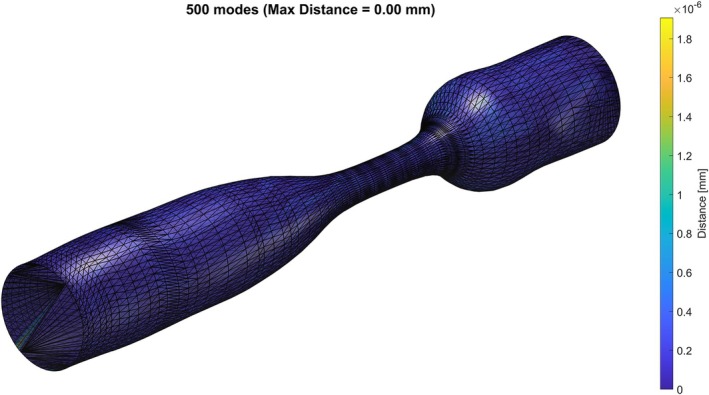
Reconstruction with 500 shape modes.

To assess reconstruction fidelity, we built a lesion approximation using 1, 5, 20, 100, and 500 PCA shape modes for a single, heuristically selected lesion chosen for its pronounced sudden tapering and high severity (79% radius reduction). Table 4 summarizes, for each deformed geometry, the maximal point‐wise error, that is, the largest vertex‐to‐surface distance, relative to the target geometry. With just five shape modes, the maximum error drops for this specific geometry to $2.7\cdot 10^{-1}$ mm (see Figure 9). Logically, adding more shape modes gradually decreases the point‐wise error further. The full set of reconstructions (Figures A1, A2, A3, A4, A5) is shown in Appendix A. We focused on these five modes to investigate whether they capture sufficient geometric variability for training the data‐driven stenosis element.

**TABLE 4:** Summary of stenotic lesion reconstructions for different numbers of PCA shape modes.

Number of shape modes	Max. point‐wise error [mm]
1	$6.8\cdot 10^{-1}$
5	$2.7\cdot 10^{-1}$
20	$1.3\cdot 10^{-2}$
100	$1.0\cdot 10^{-3}$
500	0

**FIGURE 10:**
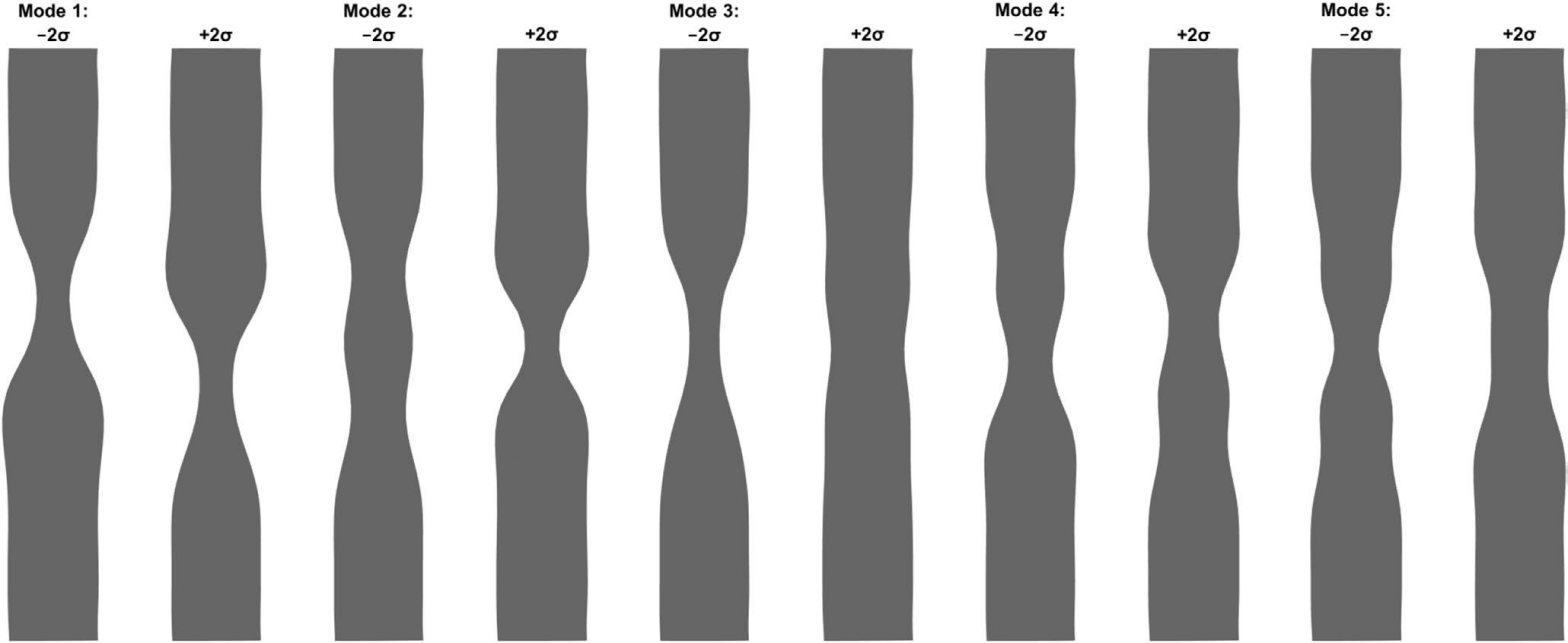
Side views of shape variations along the first five principal shape modes of deformation (±2σ). Each pair of tubes shows the shape deviation from the mean geometry at −2σ (left) and +2σ (right) for a given shape mode. From left to right in pairs: Mode 1 to Mode 5.

Figure 10 illustrates the characteristic shape variations along each of these five modes, improving the interpretability of our model. We examined the characteristic deformations associated with each shape mode to enhance our understanding of the five selected shape modes, specifically their contributions to the resulting pressure drop predictions. Mode 1 shifts the location of the minimum lumen diameter along the longitudinal lesion axis (more proximal vs. more distal); Mode 2 governs the number of local minima (single vs. double indentations); Mode 3 controls the overall severity of the stenosis (depth of constriction); Mode 4 introduces a wavy deformation and modulates whether the principal minimum is situated more distally or proximally; and Mode 5 differentiates between a trapezoidal constriction profile and a wave‐like pattern similar to that of Mode 4.

### 3.3 Large Scale CFD

**FIGURE 11:**
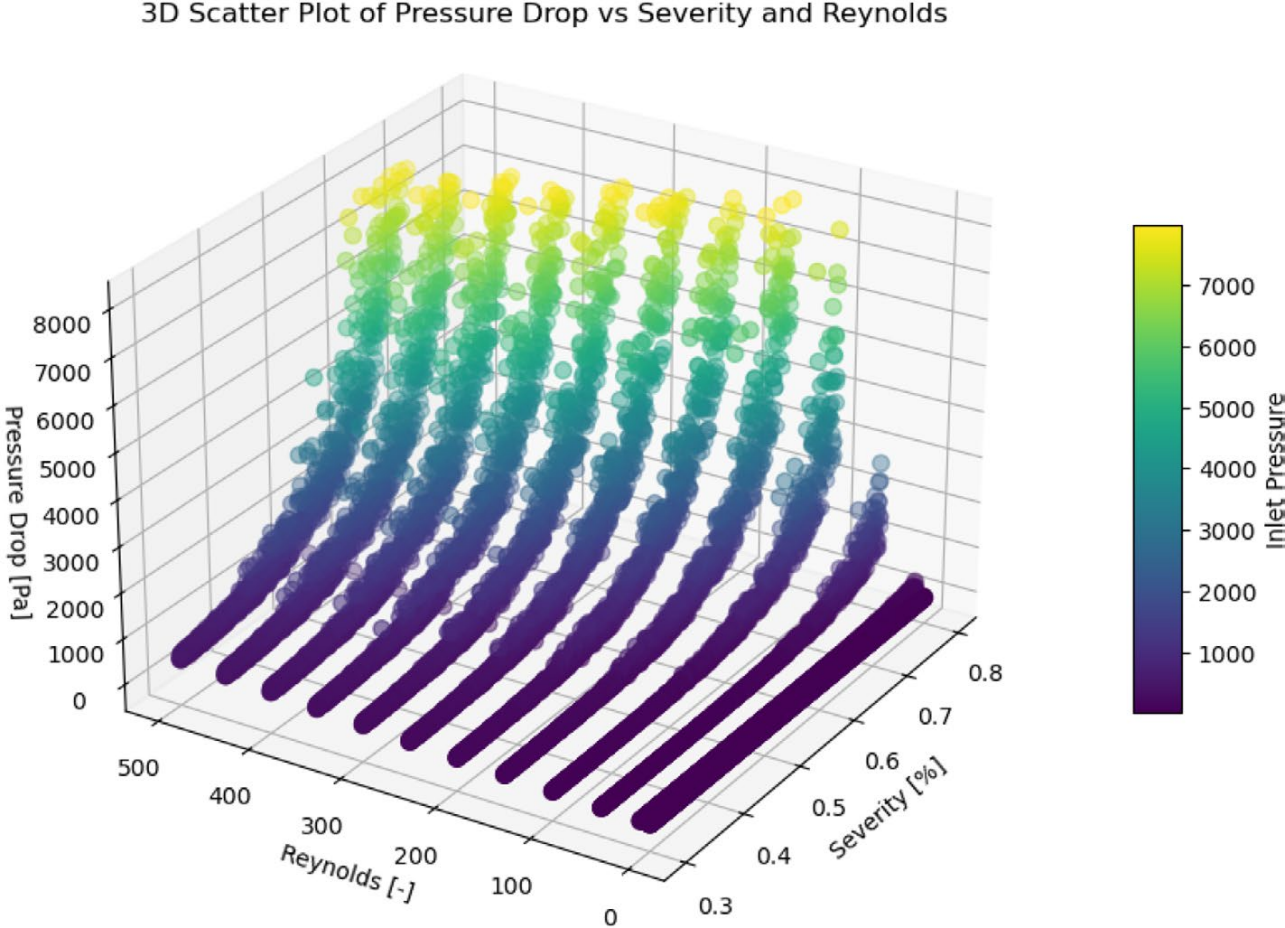
Three‐dimensional scatter plot of the CFD computed pressure drop (Δp) versus stenosis severity and Reynolds number. Points are colored by inlet pressure.

After storing each geometry's first five shape coefficients for 1024 geometries, we conducted 13,312 (1024 × 13) CFD simulations across 13 Reynolds numbers per geometry. Of the 13,312 simulations we planned, 39 simulations failed to converge due to flow‐induced instabilities, leaving 13,273 valid pressure drop data points. After excluding pressure drops greater than 60 mmHg, 11,909 pressure drops remained within the data set, as visualized in Figure 11.

As shown in Figure 11, even after applying our filter, the CFD dataset still provides continuous, “gap‐free” coverage of the entire domain. Having obtained the hemodynamic metrics (pressure drop $\Delta p_{\text{sten},3D}$ and flow q), we were able to derive the loss coefficients $K_{v}$ and $K_{t}$ per geometry.

**FIGURE 12:**
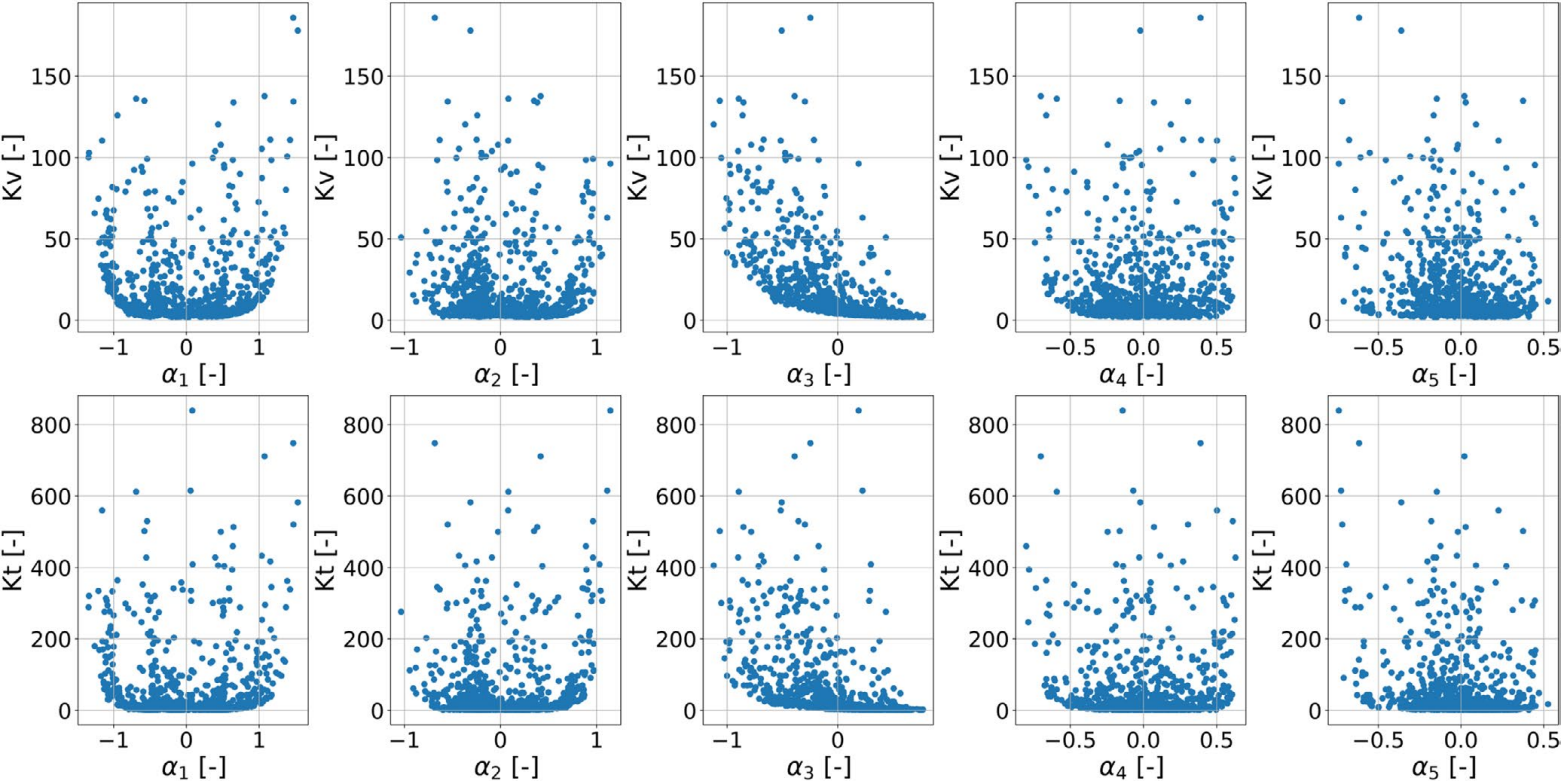
Scatterplots showing the relationship between each of the first five PCA‐derived shape modes and the loss coefficient $K_{V}$ in the top row, and the loss coefficient $K_{t}$ in the bottom row, for all samples in the training dataset.

Using linear regression, per geometry, the $K_{v}$ and $K_{t}$ are predicted, following Section 2.3.4. The geometry‐specific $K_{v}$ and $K_{t}$ are matched with their respective shape coefficients $\alpha_{i}$, forming the dataset to train the data‐driven geometry‐aware lumped arterial stenosis model. Figure 12 shows the relationship between the shape modes $\alpha_{i}$ and the $K_{v}$ and $K_{t}$ terms.

As illustrated in Figure 12, the third shape mode exhibits a negative correlation, the first shape mode displays a somewhat quadratic (U‐shaped) relationship, and the remaining modes show no clear discernible patterns. For the first shape mode, in practical terms, this means that large positive or negative deformations in the first shape mode both lead to higher loss coefficients, whereas small (near‐zero) deformations correspond to lower loss coefficients. Figure 10 reveals that when the first shape coefficient $\alpha_{1}$ approaches +1, the stenosis intensifies distally, whereas as $\alpha_{1}$ approaches −1 it intensifies proximally. This symmetric, quadratic dependence means that moving $\alpha_{1}$ toward either extreme amplifies the narrowing at the corresponding end of the lesion, which in turn drives up the values of $K_{v}$ and $K_{t}$. For the third shape mode, the negative correlation between the loss coefficients and the third shape mode can be attributed to the fact that the third shape mode mainly controls the magnitude of narrowing (as was also shown in Figure 10). Hence, the higher the constriction in the lesion, the higher the $K_{v}$ and $K_{t}$.

### 3.4 Training of the Data‐Driven Model

Both GPR models for loss coefficients $K_{v}$ and $K_{t}$ were trained with Optuna to search for the optimal hyperparameters. The optimal hyperparameters found by Optuna are listed in Table 5.

**TABLE 5:** Optuna‐optimized hyperparameters for the two GPR models for both loss coefficients.

Model	Polynomial degree	Use natural‐log transform	Kernel	ν	C	$\sigma^{2}$
$K_{v}$	1	True	Matern	1.5	$5.5\times 10^{1}$	$6.1\times 10^{-2}$
$K_{t}$	2	True	Matern	0.5	$9.3\times 10^{4}$	$1.4\times 10^{-4}$

The difference in Polynomial degree indicates that for the loss coefficient $K_{t}$, it was best to enrich the original shape vector $\alpha =\left[\alpha_{1},\ldots ,\alpha_{N_{m}}\right]$ with all second‐order terms, namely the linear terms ${\left\{\alpha_{i}\right\}}_{i=1}^{N_{m}}$, the squared terms ${\left\{\alpha_{i}^{2}\right\}}_{i=1}^{N_{m}}$, and the pairwise interactions ${\left\{\alpha_{i}\alpha_{j}\right\}}_{1\leq i<j\leq N_{m}}$. In contrast, for the loss coefficient $K_{v}$, such higher‐order effects were negligible, only the raw shape coefficients $\alpha_{i}$ without any polynomial augmentation were used.

### 3.5 Model Evaluation

After training the two GPR models $K_{v}\left(\alpha \right)$ and $K_{t}\left(\alpha \right)$, the loss coefficient model $K_{v}$ achieved an $R_{y=x}^{2}$ of 0.94 with an RMSE of 7 [−] on the 10% test set geometries, while the loss coefficient model $K_{t}$ achieved an $R_{y=x}^{2}$ of 0.82 with an RMSE of 45 [−]. When these loss coefficient predictors were used in the geometry‐aware stenosis model (Equation 21), the pressure drop predictions on the test set had an $R_{y=x}^{2}$ of 0.93 and an RMSE of 423 Pa.

**FIGURE 13:**
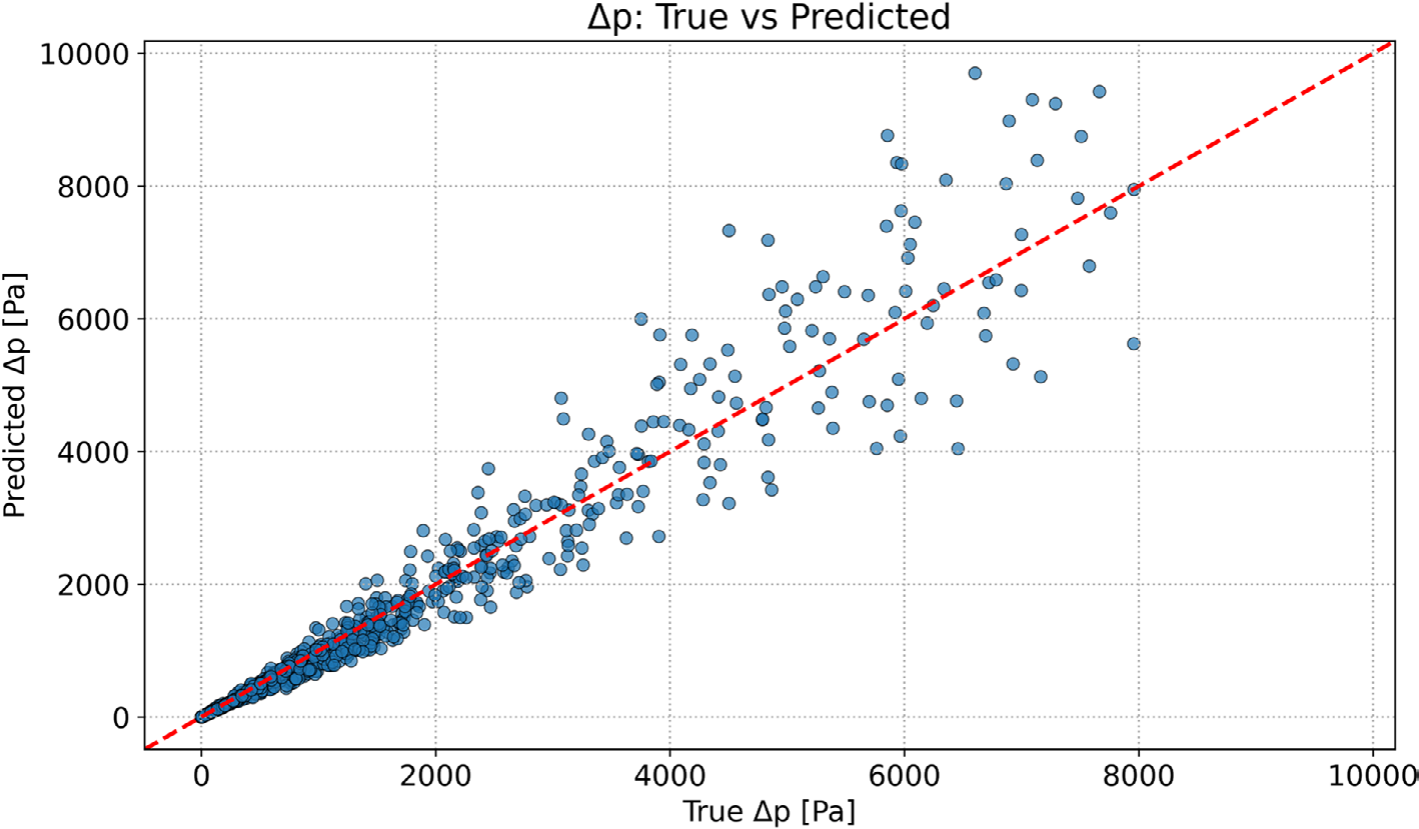
Comparison of predicted versus actual pressure drop Δp for the hold‐out test set. Each marker corresponds to one geometry, and the dashed line denotes perfect agreement (y=x).

Figure 13 shows a scatter plot of predicted versus actual pressure drops (calculated with CFD) for the test set. Especially at low values of Δp, the data points cluster tightly around the identity line y=x, indicating excellent agreement between predicted and actual pressure drops. As Δp increases, the scatter around the identity line widens, reflecting greater variability in the model's predictions at higher pressure drops.

Table 6 shows an overview of all the performance metrics for the $K_{v}$, $K_{t}$, and combined Δp models. The mean predictive standard deviations ($\sigma_{K_{v}}=1.71\;\left[-\right]$, $\sigma_{K_{t}}=13.26\;\left[-\right]$, and $\sigma_{\Delta p}=153.97\;\mathrm{Pa}$) are smaller than their respective RMSEs (${\text{RMSE}}_{K_{v}}=6.53\;\left[-\right]$, ${\text{RMSE}}_{K_{t}}=44.51\;\left[-\right]$, and ${\text{RMSE}}_{\Delta p}=423.46\;\mathrm{Pa}$), indicating that the Gaussian process regressors systematically underestimate their predictive uncertainty (i.e., are overconfident). As shown in Table 6, the mean predictive standard deviation for $K_{v}$ is σ=1.71 (vs. RMSE = 6.53), for $K_{t}$ is σ=13.26 (vs. RMSE = 44.51), and for Δp is σ=153.97Pa (vs. RMSE = 423.46 Pa), yielding RMSE‐to‐*σ* ratios of approximately 3.8, 3.4, and 2.8, respectively. A well‐calibrated predictive model would exhibit an RMSE‐to‐*σ* ratio close to one. The observed ratios therefore confirm that the regressors are overconfident, underestimating their uncertainty bounds.

**TABLE 6:** Performance metrics for the $K_{v}$, $K_{t}$, and combined Δp models.

Model	$R_{y=x}^{2}$	RMSE	Mean predictive σ
Kv	0.94 [−]	6.53 [−]	1.71 [−]
Kt	0.82 [−]	44.51 [−]	13.26 [−]
Δp	0.93 [−]	423.46 Pa	153.97 Pa

### 3.6 Data‐Driven Stenosis Model Performance Comparison

#### 3.6.1 Hemodynamic Comparison

**FIGURE 14:**
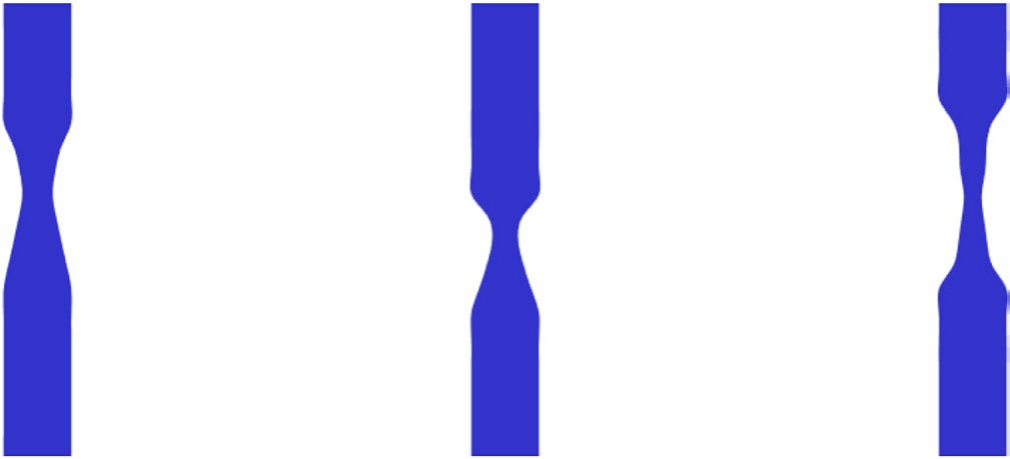
Cross‐sectional views of the three irregular stenotic lesions used for model validation. We define them as Geometry 1 ($S_{\mathrm{sev}}=55\%$), Geometry 2 ($S_{\mathrm{sev}}=61\%$), and Geometry 3 ($S_{\mathrm{sev}}=74\%$), from left to right.

The geometry‐aware data‐driven lumped arterial stenosis model was embedded in a 1D pulse wave propagation framework and benchmarked against a 1D–3D coupled simulation, in which a high‐fidelity 3D CFD component replaced the lumped stenosis element. Due to the enhanced computational costs of the 1D–3D coupled simulation, simulations were performed for three representative geometries (see Figure 14) with the stenosis of those geometries modeled via a steady 3D CFD simulation as reference, the Heinen et al. lumped stenosis model [10], and our newly trained geometry‐aware data‐driven stenosis model.

**FIGURE 15:**
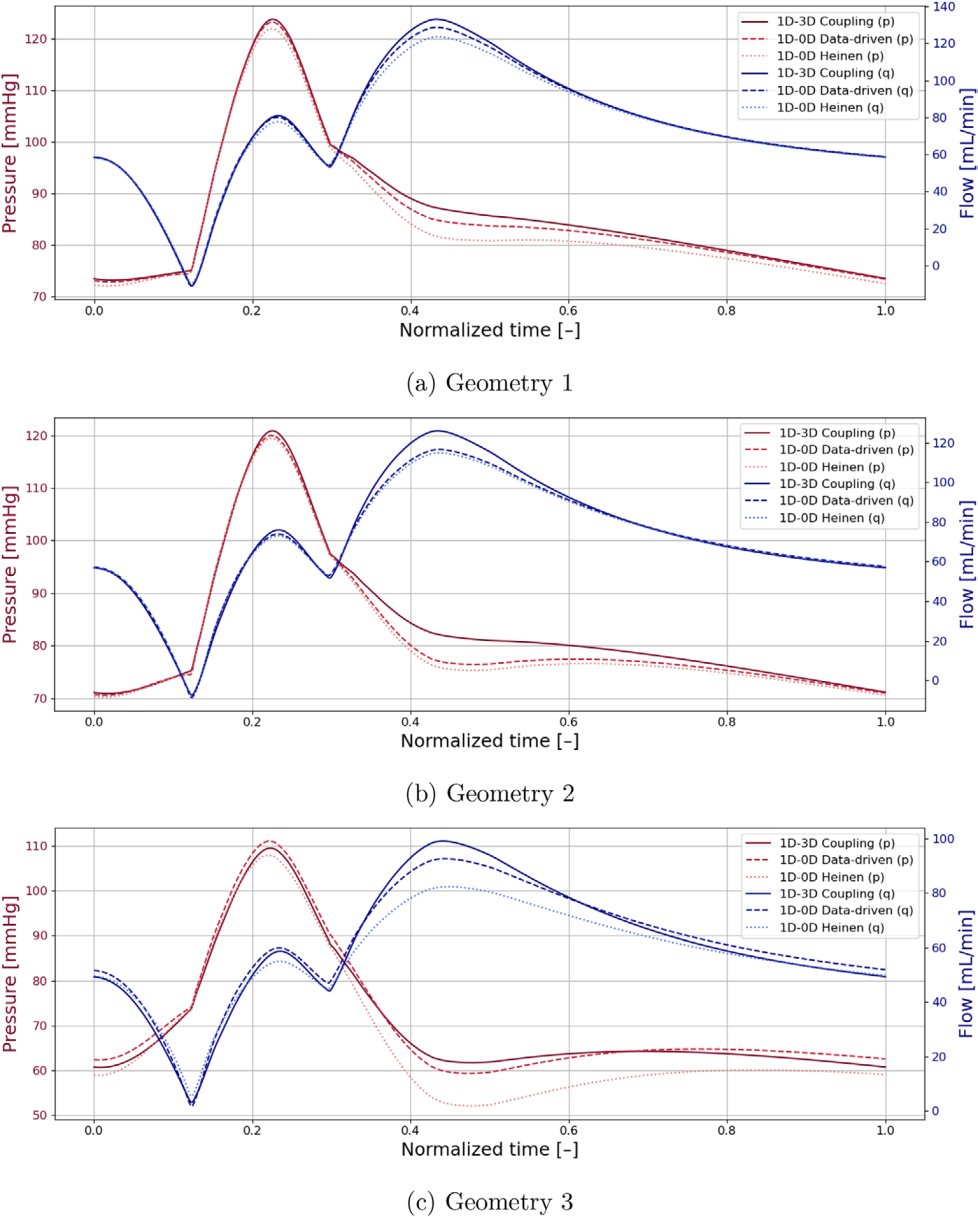
Distal‐lesion pressure and proximal‐lesion flow waveforms for three different patient‐specific geometries, each comparing different stenosis implementations: (1) A coupled 3D CFD simulation. (2) Our 0D geometry‐aware data‐driven stenosis model. (3) The 0D lumped‐parameter stenosis model of Heinen et al. [10].

The resulting pressure and flow waveforms per simulation for each geometry are depicted in Figure 15. When comparing the pressure and flow waveforms obtained with our new data‐driven stenosis element to those generated by the lumped stenosis element of Heinen et al., and benchmarking both against the high‐fidelity 1D–3D coupled simulations, it becomes clear that the data‐driven model more closely reproduces the 1D–3D results. This improvement is most pronounced for Geometry 3, whose shape deviates substantially from the idealized cosinusoidal profile assumed by the Heinen model. Nevertheless, even for the more cosinusoidal Geometries 1 and 2, the data‐driven element consistently yields waveforms that lie nearer to the reference 1D–3D solutions, demonstrating its superior accuracy and robustness across a range of stenosis geometries.

#### 3.6.2 FFR Prediction Comparison

**FIGURE 16:**
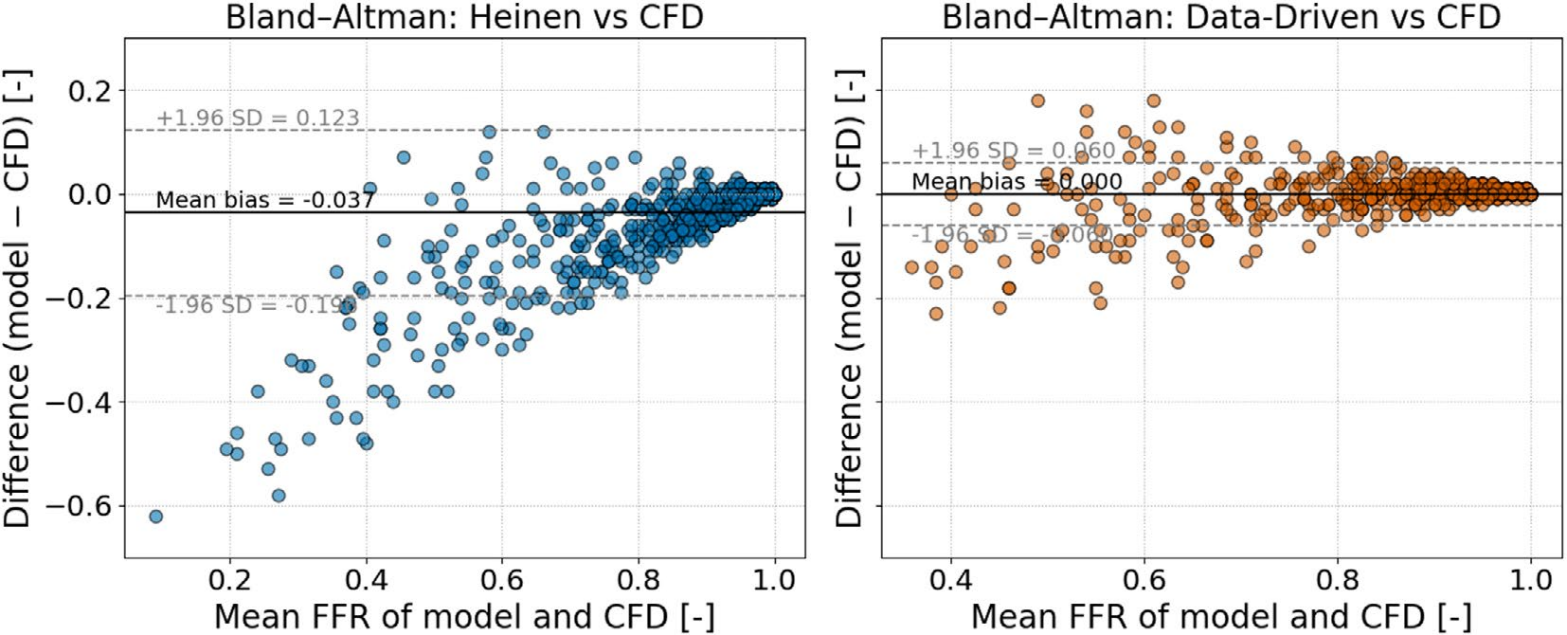
Bland–Altman comparison of FFR predictions from the Heinen et al. model (left) and the data‐driven model (right) against the CFD‐derived ground truth. The mean bias and ±1.96 standard deviation limits are shown as solid and dashed lines, respectively.

To assess whether the model can be used as a non‐invasive estimator of the FFR, the FFR was computed, via Equation (25), using the newly trained data‐driven model and the approach of Heinen et al. [10]. These FFR estimates were then compared against the CFD‐derived ground‐truth values (${\mathrm{FFR}}_{\mathrm{CFD}}$). Both models predicted the FFR within 0.02 of the CFD‐derived ground truth in about 60% of test cases. An additional 3% of cases were correctly predicted only by the Heinen et al. model, giving it an overall accuracy of about 62%. In approximately 20% of cases, only the geometry‐aware data‐driven model correctly predicted the pressure drop, resulting in an overall accuracy of about 80% for the data‐driven model. Only approximately 18% of cases were mispredicted by both models by more than 0.02, so the data‐driven model erred in just 20% of cases compared to 38% for the Heinen et al. model. Additionally, Figure 16 presents a Bland–Altman plot summarizing the performance of each model.

The Bland–Altman analysis further highlights these differences: the Heinen et al. model showed a mean bias of −0.04 with limits of agreement from about −0.20 to +0.12, indicating a slight underestimation of FFR. The data‐driven model, however, exhibited a negligible bias (0.00) and much narrower limits (−0.06 to +0.06), reflecting closer agreement with the CFD ground truth and reduced variability. Overall, the Bland–Altman plots confirm the improved accuracy and consistency of the data‐driven model.

**FIGURE 17:**
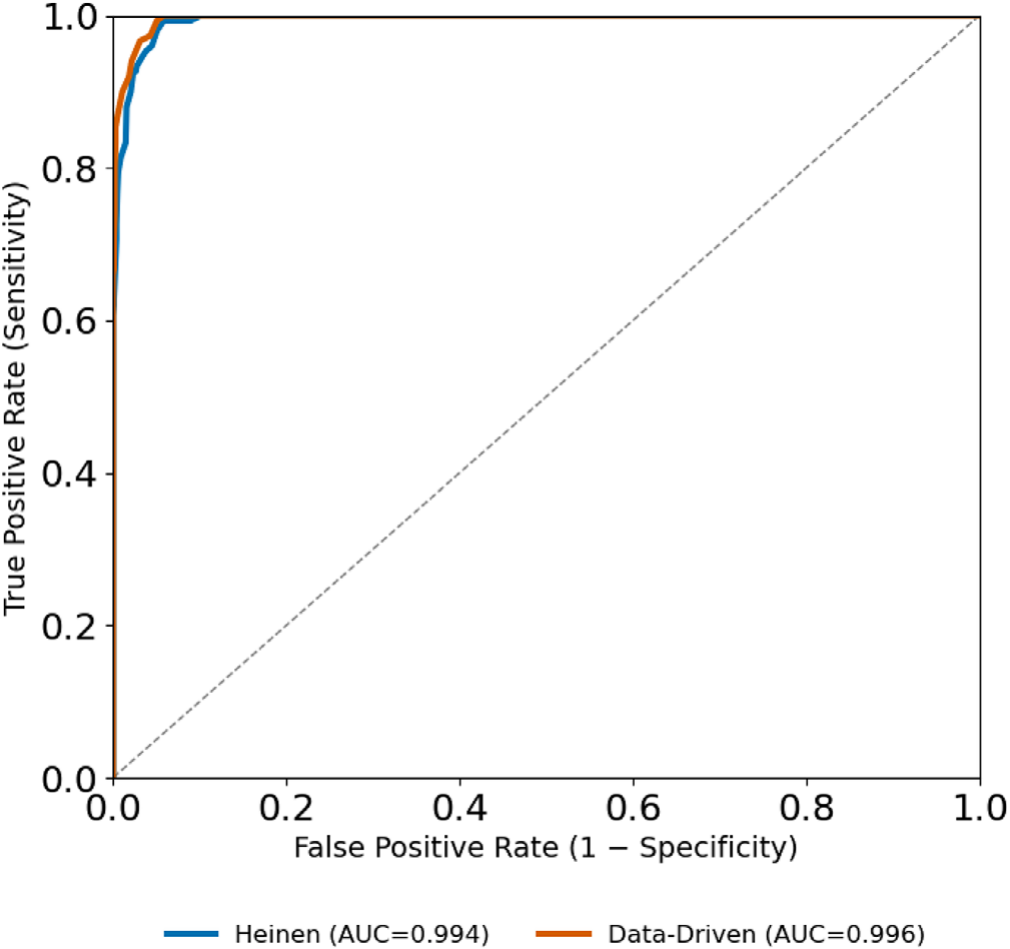
Receiver operating characteristic (ROC) curves comparing the classification performance of the Heinen et al. model and the geometry‐aware data‐driven model relative to the CFD‐derived ground truth at the clinical FFR threshold of 0.8.

Since FFR guides the decision for invasive treatment [7], we assessed each model's binary classification performance at the clinical threshold of FFR=0.8 (i.e., FFR≤0.8 vs. FFR>0.8). Receiver operating characteristic (ROC) analysis was performed (see Figure 17) to quantify the models their ability to discriminate between ischemic (FFR≤0.8) and non‐ischemic cases (FFR>0.8). Both models achieved excellent classification performance, with areas under the ROC curve (AUC) of 0.994 for the Heinen et al. model and 0.996 for the data‐driven model. The data‐driven model demonstrated a small but not statistically significant improvement in AUC (ΔAUC = 0.002, p=0.055, DeLong test for correlated ROC curves), indicating no meaningful improvement in diagnostic performance at the clinical decision boundary.

Overall, the ROC analysis confirms that both models can accurately identify clinically significant ischemia, with the data‐driven model offering slightly improved discriminative performance.

Lastly, for the three geometries in Section 3.6.1, we computed FFR by deriving the proximal (aortic) and distal pressure from the 1D pulse wave propagation model using three distinct approaches: 1D coupled to full 3D CFD simulations, 1D plus the data‐driven stenosis model using each geometry's shape modes, and 1D plus Heinen's lumped model parameterized by the geometry's geometric characteristics. Table 7 summarizes the resulting FFR values:

**TABLE 7:** FFR predictions of the 1D pulse wave propagation model for three stenosis model set‐ups: 1D–3D CFD (reference), geometry‐aware data‐driven, and Heinen et al. [10].

Geometry	1D–3D CFD	1D and data‐driven	1D and Heinen
1	0.94	0.94	0.92
2	0.91	0.90	0.89
3	0.77	0.78	0.73

As shown in Table 7, the data‐driven model exactly reproduces the 1D–3D CFD reference for Geometry 1 (0.94 vs. 0.94) and slightly underestimates it for Geometry 2 (0.90 vs. 0.91). For the most severe and least idealistic stenosis (Geometry 3), it predicts 0.78, which is notably closer to the CFD result (0.77) than the Heinen et al. model (0.73), demonstrating improved agreement across all stenosis severities.

## 4 Discussion

In this study, we developed a geometry‐aware, data‐driven lumped arterial stenosis model by training it on high‐fidelity CFD simulations combined with geometry‐descriptive shape modes. This approach enables fast geometry‐specific estimation of loss coefficients, which were integrated into a commonly used pressure drop formula, enhancing the predictive capabilities of lumped stenosis models for irregular and thus more realistic lesions.

### 4.1 Model Performance

Our assumption that five shape modes are sufficient for accurately predicting pressure drops was confirmed by our findings, demonstrating that only a handful of modes effectively capture the geometric variability that drives hemodynamic predictions. Although incorporating additional shape modes (e.g., 15 modes capturing 99.6% of the total variability) might in principle improve descriptive power, we observed no measurable benefit on the geometries and the prediction in pressure drop in this study. In fact, the extra modes appear to contribute more noise than signal, ultimately degrading the regression models' ability to fit the data accurately. Introducing greater variability into the geometric dataset, particularly with real lesions, may necessitate additional shape modes to capture nuanced geometric features.

The geometry‐aware data‐driven stenosis model, when applied to the synthetic stenotic geometries, demonstrated increased performance compared to the Heinen model. Although Table 6 reveals that both Gaussian process regressors are somewhat overconfident, as their average predictive uncertainties (σ) are smaller than the corresponding RMSEs (indicating they underestimate their uncertainty bounds). Although the Δp predictions remain imperfect, exhibiting moderate generalization and slight overconfidence, they nonetheless represent a significant improvement in comparison with the stenosis model of Heinen et al. [10]. Our previous work showed that the Heinen lumped stenosis model performs suboptimally for realistic, diffuse lesions [18]. In contrast, our data‐driven stenosis model aligns far more closely with high‐fidelity 1D–3D CFD results [10], capturing the true 3D pressure and flow variations resulting from the complex geometry. This advantage is most pronounced in Geometry 3, which is characterized by a pronounced irregular, non‐cosinusoidal narrowing. In contrast, the Heinen model significantly overpredicts this pressure drop, while our SSM‐based predictions align relatively closely with the 1D–3D reference. This was further emphasized by the fact that our newly trained model significantly improves FFR prediction accuracy, correctly simulating FFR in 80% of test cases, an improvement of 18% over the Heinen model. This was also evident for our three example geometries, as depicted in Table 7. Based on clinical cut‐offs, both models accurately classified stenotic lesions as ischemia‐inducing in approximately 93% of cases; however, the data‐driven model misclassified only 2% of cases. Importantly, as a lumped parameter framework, this model retains the high computational efficiency of lumped approaches, allowing for pressure drop and thus FFR estimations that closely resemble CFD results in minutes, when integrated within the 1D pulse wave propagation framework, compared to the hours (or maybe even days) required for full 3D CFD simulations.

Several rapid surrogate modeling approaches have been proposed to accelerate pressure–drop estimation. Notably, Hu et al.'s FAST framework, Pajaziti et al.'s shape‐driven neural networks, and Itu et al.'s ML‐based FFR model all achieve dramatic speed‐ups over full 3D CFD, but each is trained and applied under a single hemodynamic condition [29, 30, 31]. Hu et al.'s [29] FAST framework calibrates six global correction factors using only steady state maximal hyperemic CFD simulations, optimizing these factors against 20 patient‐specific 3D CFD runs at maximal hyperemia, and then applies the same factors to all new geometries under similar hyperemic conditions. Similarly, Pajaziti et al. [31] have created a shape mode‐driven deep neural network that is trained and validated exclusively at a single inlet velocity of 1.3 m/s (peak systolic aortic flow), limiting its predictive scope to that fixed flow regime. Likewise, Itu et al. [30] train a random‐forest model on a synthetically generated database of 12,000 coronary anatomies—extracting handcrafted geometric features and labeling them via reduced‐order CFD—to predict FFR values along the vessel centerline, but only under hyperemic conditions. By contrast, our lumped stenosis model learns two lesion‐specific loss coefficients (linear and non‐linear) for each geometry by fitting against a continuum of CFD simulations spanning resting through hyperemic Reynolds numbers. While this Reynolds‐number sweep ensures that the model inherently captures varying physiological flow states and eliminates the need for case‐by‐case recalibration, its predictive accuracy decreases more substantially at the highest Reynolds numbers. This reduced accuracy arises primarily from two factors. First, the training dataset contains comparatively few samples in this regime, since such extreme flow conditions occur only in cases of severe stenosis combined with high flow rates. Second, at high Reynolds numbers the flow field becomes increasingly complex, with stronger flow disturbances, recirculation zones, and nonlinear pressure–velocity interactions that are more difficult to capture with a lumped representation. Consequently, the model has less exposure and reduced representational capacity for these highly nonlinear behaviors, leading to larger deviations in predicted pressure drops. Nonetheless, the model preserves a simple, interpretable structure, similar to Heinen et al. [10], while remaining applicable across a broad range of flow conditions, in contrast to single‐point surrogate models. In this study, we could have opted to include Reynolds as another input for our Gaussian process regressors to make the model even more versatile. However, it was found that this did not yield an improved predictive outcome regarding the pressure drop, and it was therefore omitted from this paper. The major benefit of solely training on shape modes is that new lesion shapes can be accommodated instantly by projecting them into the same low‐dimensional shape space. This facilitates the integration of new, possibly real, lesion shapes with relative ease.

Moreover, by learning to predict the instantaneous pressure drop Δp across a stenosis rather than the FFR directly, our approach remains aligned with the formulation of pressure losses in hemodynamics. The model retains the conventional form

(26) $$\Delta p\;=\;K_{v}\;\left(\frac{8\;\mu \;l_{s}}{\pi \;a_{0}^{4}}\;q\right)\;+\;K_{t}\;\left(\frac{1}{2}\;\rho {\left(\frac{q}{\pi a_{0}^{2}}\right)}^{2}\right),$$

in which the learned loss coefficients $K_{v}$ and $K_{t}$ scale the linear and nonlinear loss terms, respectively. While these coefficients are determined from data and therefore not directly interpretable as physical quantities, the preserved equation structure remains physically motivated and compatible with the principles of hemodynamics. This formulation ensures straightforward integration with the 1D pulse wave propagation model, enabling direct comparison with conventional lumped‐parameter formulations.

Decoupling pressure‐drop estimation from the downstream FFR computation further allows targeted analysis of how geometric perturbations, captured via shape mode decomposition, affect the fitted values of the loss coefficients $K_{v}$ and $K_{t}$. This provides a structured means to assess the relative influence of different lesion features on the pressure drop. We note, however, that a direct end‐to‐end prediction of FFR could, in principle, offer greater modeling flexibility and potentially higher predictive accuracy. Nonetheless, the present formulation was chosen to ensure compatibility with the 1D PWPM framework and to maintain a physically motivated model structure that facilitates modular integration and interpretation.

Overall, this study serves as proof of concept, demonstrating the promise of a data‐driven, geometry‐aware lumped arterial stenosis model while laying a solid, scalable foundation for future validation, feature selection, and methodological refinement on the path to clinical application.

### 4.2 Model Limitations

While our geometry‐aware, data‐driven stenosis model outperforms the classic Heinen formulation, several limitations remain. First, although our synthetic lesions span a range of constriction severities beyond Heinen's cosinusoidal profiles, they remain to some extent idealized and axisymmetric, lacking features such as eccentric plaques, vessel curvature, side‐branch interactions, and calcified nodules common in clinical cases. Second, training exclusively on CFD simulations of these synthetic geometries does not expose the model to physiological factors, like vessel wall compliance, pulsatile flow, and heterogeneous plaque composition, that influence in vivo pressure drop behavior.

However, by fitting our low‐dimensional template of shape modes to new, for example patient‐derived, geometric lesions, our framework establishes a truly extensible foundation for modeling increasingly complex and diffuse stenoses without altering the core methodology. This template‐fitting paradigm offers a clear roadmap for iterative enhancement: one simply augments the shape basis by training on larger, more comprehensive datasets to capture multi‐focal, eccentric, or even curved lesion morphologies. Naturally, expanding the feature space introduces its own challenges: template‐to‐target registration becomes more demanding, an excessive number of modes can lead to overfitting or inject high‐frequency noise that may even prevent convergence of the regression model describing the pressure–flow relationship, and alignment accuracy can degrade.

Although the pressure drop can be computed across various flow regimes, it should be noted that the present model is limited to steady‐state conditions and does not account for dynamic pressure variations arising from transient acceleration or deceleration of the fluid, as it was solely trained using steady boundary conditions. Although not in the scope of this study, future research should extend the model to incorporate unsteady effects to improve its applicability in dynamic simulations.

Moreover, the FFR values derived from the CFD and lumped models used for Figures 16 and 17 are based on fixed flow conditions and therefore neglect pulsatile and inertial effects. However, previous transient simulations have shown these effects to be minor under similar flow regimes [18], supporting the use of steady‐flow FFR as a valid surrogate for model comparison.

Another limitation of the current study is the assumption that the loss coefficient $K_{v}$ remains constant across all Reynolds numbers. While this approach is consistent with the method of Heinen et al. [10], it may not hold for complex non‐idealized three‐dimensional geometries where local flow patterns and secondary effects can influence the effective contribution of the linear term [32]. This simplification could partly explain the reduced model accuracy observed at higher Reynolds numbers, suggesting that future work should consider formulations allowing Reynolds‐dependent or coupled linear and nonlinear effects.

Finally, we note that the quasi‐steady formulation of Equation (26), while effective for steady or smoothly varying flow conditions, may be less suited to the highly pulsatile and spatially diffuse flow environments characteristic of coronary arteries [33]. In coronary hemodynamics, local accelerations and decelerations due to myocardial motion and complex vessel geometry can produce transient pressure losses that are not captured by the present quasi‐steady model. However, it should be noted that the 1D–3D coupled simulations used for model comparison in this study also employ a steady 3D formulation, and in our previous work [18], we demonstrated that such a steady formulation is sufficient to accurately assess time‐averaged pressure drops and FFR, the primary outputs of interest here. Therefore, the quasi‐steady assumption adopted in this study is appropriate within this context, though it may not capture transient effects or instantaneous flow–pressure dynamics. Future work should extend the formulation to incorporate unsteady terms (e.g., proportional to $\frac{\mathrm{dQ}}{\mathrm{dt}}$) or utilize pulsatile flow data to improve predictive fidelity in dynamic coronary simulations.

Lastly, it should be noted that we did not extract multiple $K_{t}$ values as functions of $\alpha_{i}$ and Reynolds number. Although incorporating Reynolds number as an input for predicting $K_{t}$ improved the fit of the nonlinear *K*_*t*_‐prediction model (see Section 2.4), the improvement was marginal (approximately 6%) and did not enhance the accuracy of the resulting pressure drop predictions. Thus, to maintain the simpler nonlinear regression model input space, we retained only one $K_{t}$ value per geometry.

### 4.3 Extrapolatability and Future Implementations

The logical next step would be to validate the data‐driven, geometry‐aware stenosis model using real patient geometrical data and confirm its performance under real‐world conditions. In practice, standard angiography provides only two‐dimensional projections that can underestimate lesion complexity. Advanced modalities, such as CT or IVUS, offer three‐dimensional reconstructions, significantly enhancing geometric characterization and model accuracy [34], albeit these processes are not simplistic either. Properly validating this geometry‐aware stenosis model would require training the statistical shape model on a comprehensive dataset encompassing the full spectrum of possible lesion geometries. Scaling factors, as used by Verstraeten et al. [13], will ensure that the simplistic tube template geometry can be deformed easily to accommodate lesions of varying sizes. Specialized models could potentially account for more complex scenarios, such as closely located multiple lesions, curved vessels, or bifurcations. The framework could be extended to capture a broader range of lesion geometries by enriching the shape modes with additional geometric information, thus increasing the number of modes required to represent sufficient anatomical variability.

Additionally, the framework could be extended by including lesion‐specific terms in Equation (21). Schrauwen et al. [35] demonstrated that curvature alone accounts for approximately 18% of the total pressure drop at Re = 250, highlighting the value of incorporating dedicated geometric terms. Vessel bifurcations exhibit unique pressure–flow relationships [36] that could be modeled by constructing a bifurcation‐specific statistical shape model (using a bifurcation‐specific template) and training a dedicated lumped model. Similarly, curvature effects could be addressed by introducing a curvature correction term. This could be done by virtually straightening curved vessel segments and comparing the pressure drops before and after straightening. The resulting difference could then be used as a correction to the straight lesion pressure drop prediction, ensuring curvature effects are properly accounted for.

To ensure that such extensibility yields reliable and generalizable predictions, future work must therefore strike a balance between geometric expressiveness and model robustness. The question is: How much detail must be captured to ensure good pressure drop predictions? For example, by integrating regularization schemes or adopting hierarchical shape modeling [37, 38, 39]: start with a low‐order basis of global deformation modes (e.g., overall constriction severity and length), then add intermediate modes (e.g., regional eccentricity, curvature), and only include fine‐scale modes (e.g., surface roughness or calcified nodules) when needed to control capacity and minimize overfitting.

As coronary angiography is the most‐prevalent imaging modality used for coronary artery disease diagnostic, a potential clinical workflow could involve initially evaluating, through visual inspection of angiograms, whether the lesion exhibits relatively simple geometric characteristics, such as cosinusoidal shapes, for which the Heinen model would provide the most efficient pressure drop and thus FFR estimate [18]. However, if the lesion geometry is more complex, the more complex SSM framework proposed in this study could be employed for a more accurate, still relatively fast, geometry‐specific prediction of pressure drop and subsequent FFR estimation. However, this would require more advanced imaging modalities such as CT and IVUS.

## 5 Conclusion

This study presents a fast, geometry‐aware, data‐driven lumped arterial stenosis model that combines statistical shape modes with high‐fidelity CFD data to estimate both linear and non‐linear pressure‐drop coefficients. The model could easily be implemented within a 1D pulse wave propagation model, and by using the shape coefficients, the pressure drop predictions more closely resembled those of 3D CFD simulations than another state‐of‐the‐art methodology, especially in cases of irregular lesions. Critically, only five shape modes were needed to capture the essential geometric variability, underscoring the method's efficiency. Although validated on a synthetic lesion cohort, the framework is readily extensible through the addition of further shape modes, curvature terms, or lesion‐specific sub‐models, and awaits further validation on patient‐derived geometries. With further refinement and real‐world testing, this model could serve as the base for more rapid, geometry‐tailored FFR assessment in routine practice.

## Funding

This work was supported by the European Union's Horizon 2020 Research and Innovation Programme through the Research and Innovation Actions “In Silico World: Lowering Barriers to Ubiquitous Adoption of In Silico Trials”, 101016503, and “SIMCor: In‐Silico testing and validation of Cardiovascular IMplantable devices”, 101017578. Polish High‐Performance Computing Infrastructure PLGrid (HPC Center: ACK Cyfronet AGH) PLG/2024/017022, PLG/2024/017108.

## Ethics Statement

The authors have nothing to report.

## Conflicts of Interest

The authors declare no conflicts of interest.

## Data Availability

The data that support the findings of this study are available from the corresponding author upon reasonable request.

## Appendix A Detailed Mode‐by‐Mode Reconstructions

To illustrate the effect of truncating the PCA‐derived shape basis, we reconstructed the stenotic lesion geometry using 1, 5, 20, 100, and 500 modes. Figures A1, A2, A3, A4, A5 show each reconstruction, shaded by the pointwise distance to the target geometry (wireframe).

It is clear from Figure A1 that one mode is insufficient to capture the lesion‐induced deformation (max. error ≈ 0.6 mm).

Using five modes reduces the maximum point‐wise error to about 0.25 mm, considerably improving the fit while keeping complexity low.

With 20 modes the diverging portion is faithfully reproduced (max. error ≈ $10^{-2}$ mm).

At last, Figures A4 and A5 demonstrate that beyond 100 modes the reconstruction error becomes negligible.
